# Novel *Rbfox2* isoforms associated with alternative exon usage in rat cortex and suprachiasmatic nucleus

**DOI:** 10.1038/s41598-017-10535-3

**Published:** 2017-08-30

**Authors:** L. M. M. Partridge, D. A. Carter

**Affiliations:** School of Biosciences, Cardiff University, Cardiff, UK

## Abstract

Transcriptome diversity in adult neurons is partly mediated by RNA binding proteins (RBPs), including the RBFOX factors. RBFOX3/NeuN, a neuronal maturity marker, is strangely depleted in suprachiasmatic nucleus (SCN) neurons, and may be compensated by a change in *Rbfox2* expression. In this study, we found no superficial changes in *Rbfox2* expression in the SCN, but mRNA population analysis revealed a distinct SCN transcript profile that includes multiple novel *Rbfox2* isoforms. Of eleven isoforms in SCN and cerebral cortex that exhibit exon variation across two protein domains, we found a 3-fold higher abundance of a novel (‘−12–40’) C-terminal domain (CTD)-variant in the SCN. This isoform embraces an alternative reading frame that imparts a 50% change in CTD protein sequence, and functional impairment of exon 7 exclusion activity in a RBFOX2-target, the L-type calcium channel gene, *Cacna1c*. We have also demonstrated functional correlates in SCN gene transcripts; inclusion of *Cacna1c* exon 7, and also exclusion of both NMDA receptor gene *Grin1* exon 4, and *Enah* exon 12, all consistent with a change in SCN RBFOX activity. The demonstrated regional diversity of *Rbfox2* in adult brain highlights the functional adaptability of this RBP, enabling neuronal specialization, and potentially responding to disease-related neuronal dysfunction.

## Introduction

Alternative gene splicing is a major determinant of neuron-specific function^1^, affecting both individual proteins, and ultimately, protein interaction networks^2^. Multiple factors contribute to alternative splicing, and of these, RNA binding proteins (RBPs) are known to be important determinants of cell-specific splicing^3^. In neurons, one family of RBPs, the RNA-binding FOX homolog (RBFOX) proteins^4^, exert widespread effects on neuronal gene splicing^5–7^. However, the functional repertoire of RBFOX proteins remains undefined, partly because the three proteins in this family, RBFOX1 (A2BP1), RBFOX2 (Rbm9) and RBFOX3 (NeuN) appear to have both common and distinct properties^4, 8^. *Rbfox* genes are differentially expressed; *Rbfox1* in neurons, heart and muscle, *Rbfox2* more widely in neurons, muscle and multiple progenitor cells, whereas *Rbfox3/*NeuN is famously neuron-specific^4^. In the adult brain, all three RBFOX proteins are widely, but again, differentially expressed across different regions^9^. RBFOX proteins share a consensus RNA binding sequence (U)GCAUG that interacts with a conserved RNA recognition motif (RRM) within a central region of all RBFOX proteins. Position-dependent binding of RBFOX proteins relative to alternative exons can mediate either exon inclusion (downstream intronic binding), or exon skipping (upstream intronic binding)^7, 10^. N-terminal (NTD) and C-terminal (CTD) domains of the RBFOX proteins are important additional determinants of cell-specific function^11–13^.

Our work demonstrating an absence of the ‘pan-neuronal’ RBP, RBFOX3/NeuN, from the majority of SCN neurons^14^ is indicative of significant changes in splicing regulation within this hypothalamic population that may underlie specific functional requirements of this integrative centre in the brain^15^. Previous studies have provided evidence of SCN-selective gene splicing^16–18^, but the mechanistic basis of these differences is unknown. In the current study, we have now addressed one aspect of splicing regulation in this brain region by analysing SCN expression and activity of the *Rbfox3*/NeuN sister gene, *Rbfox2*.

*Rbfox2* was selected for this study because the RBFOX3/NeuN-negative^14^, SOX2-positive^19^ phenotype of SCN neurons is consistent with relative neuronal immaturity. *Rbfox2* is a good candidate because unlike the other Rbfox genes, it is expressed early in neuronal development, and in particular, is found in early post-mitotic neurons in addition to mature neurons^9, 20–22^. Also, recent studies have shown that cortical *Rbfox2* expression decreases in the adult brain (in general), whereas *Rbfox1* has the opposite temporal profile^23^. Therefore, *Rbfox2* appears a likely candidate for possible expression in ‘immature’, RBFOX3-depleted, adult SCN neurons. Currently, *Rbfox2* is known to have a specific role in cerebellar development^24^, but roles of *Rbfox2* in the adult brain are undefined.

## Results

### RT-PCR identification of rat brain *Rbfox2* cDNA

**Figure 1:**
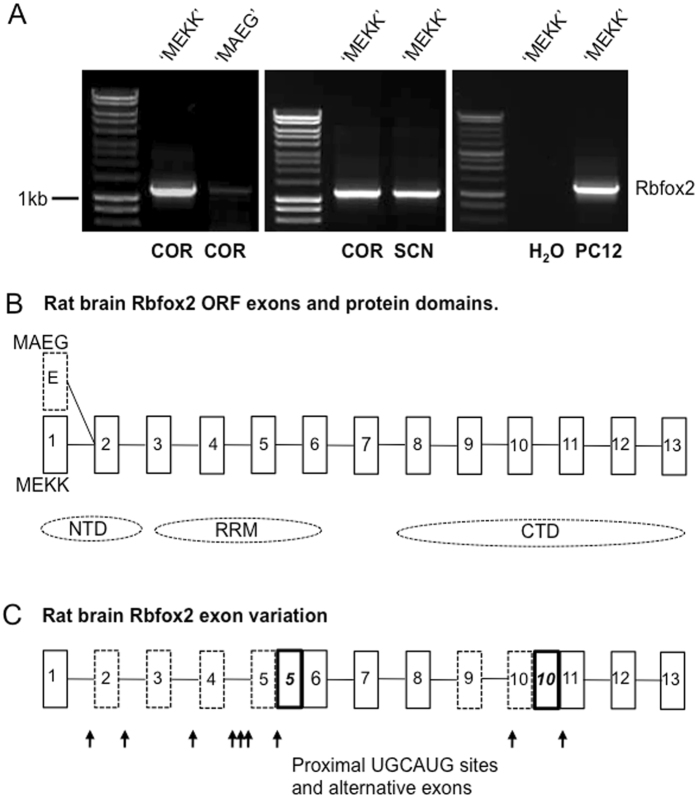
Rbfox2 is highly expressed in rat brain, and multiple isoforms are derived from a multi-exon transcript that includes a ‘MEKK’ ATG start. (**A**) Representative agarose gel electrophoresis images of RT-PCR analysis using forward primers directed against two different Rbfox2 start sites represented as ‘MEKK’ and ‘MAEG’. Left: abundance of ‘MEKK’ isoforms compared with ‘MAEG’ isoforms in rat cerebral cortex (COR). Centre: similar abundance of ‘MEKK’ isoforms in cortex and suprachiasmatic nucleus (SCN). Right: abundance of ‘MEKK’ isoforms in PC12 cells. PCR product size is estimated against either Hyperladder I (left and centre) or Promega 1 kb ladder (right). (**B**) Schematic representation of cloned rat brain Rbfox2 cDNA structure showing (predicted) alternative 5′ exons and approximate coding positions of the protein domains encoded by ‘MEKK’ isoforms (N-terminal, NTD; RNA recognition motif domain, RRM; C-terminal, CTD). The ‘MAEG’ ‘exon’ (E) is shown dashed to indicate that there may be more than 1 exon in this alternative 5′ start. Exons and introns are not to scale. (**C**) Schematic representation of rat brain Rbfox2 cDNA structure showing sites of exon variation (dashed boxes), and alternative exon insertions (bold italic numbers) in cloned rat brain Rbfox2 cDNAs. Arrows indicate the location of proximal intronic (within 500 nt) UGCAUG (consensus RBFOX-binding) sequences.

In the absence of published sequence information on rat brain *Rbfox2* transcripts, PCR primers (Table S1) were based on (i) a partially annotated rat transcript (NM_001079895.1), and (ii) rat genome sequence corresponding to mouse transcripts that have an alternative translation start site (eg. NM_001286418). The primer pairs used for this analysis thereby target the two alternative ATG codons recognized in mouse and human Rbfox2 transcripts^11^, together with a full-length open reading frame with known (alternative) N-terminal, RRM, and C-terminal domains (Fig. 1). However, potential alternative 5′ and 3′ UTR sequence is not addressed using this primer design. RT-PCR analysis revealed two distinct *Rbfox2* transcripts in rat cerebral cortex but a very marked, and consistent, abundance of the ‘MEKK’ start transcript compared with the ‘MAEG’ transcript (Fig. 1A). This result was essentially identical in rat SCN (Fig. S1) and, superficially, levels of MEKK-containing transcripts were similar in SCN and cortex (Fig. 1A). Given these results, subsequent experiments were focused on MEKK-containing transcripts/protein (see protein data below), but it is recognized that these RT-PCR analyses do not exclude the expression of alternative transcripts. Initial sequence analysis of the RT-PCR-generated rat cortex MEKK transcript revealed a 1175 bp sequence that includes some 5′ and 3′ UTR sequence included in primers RBFF6 and RBFR3 (Supplemental data, SI1). Mapping this sequence to the rat genome with BLAT indicates division into 13 exons. The open reading frame (ORF) sequence of 1143 bp codes for a 381 amino acid protein that is homologous to, and highly conserved with, annotated mouse (NP_001104298.1; 99.7% identity) and human (NP_001336926.1; 98.7% identity) RBFOX2 isoforms. Of note, the predicted CTD is 100% conserved across these 3 species.

### RBFOX2 has abundant nuclear expression in SCN

**Figure 2:**
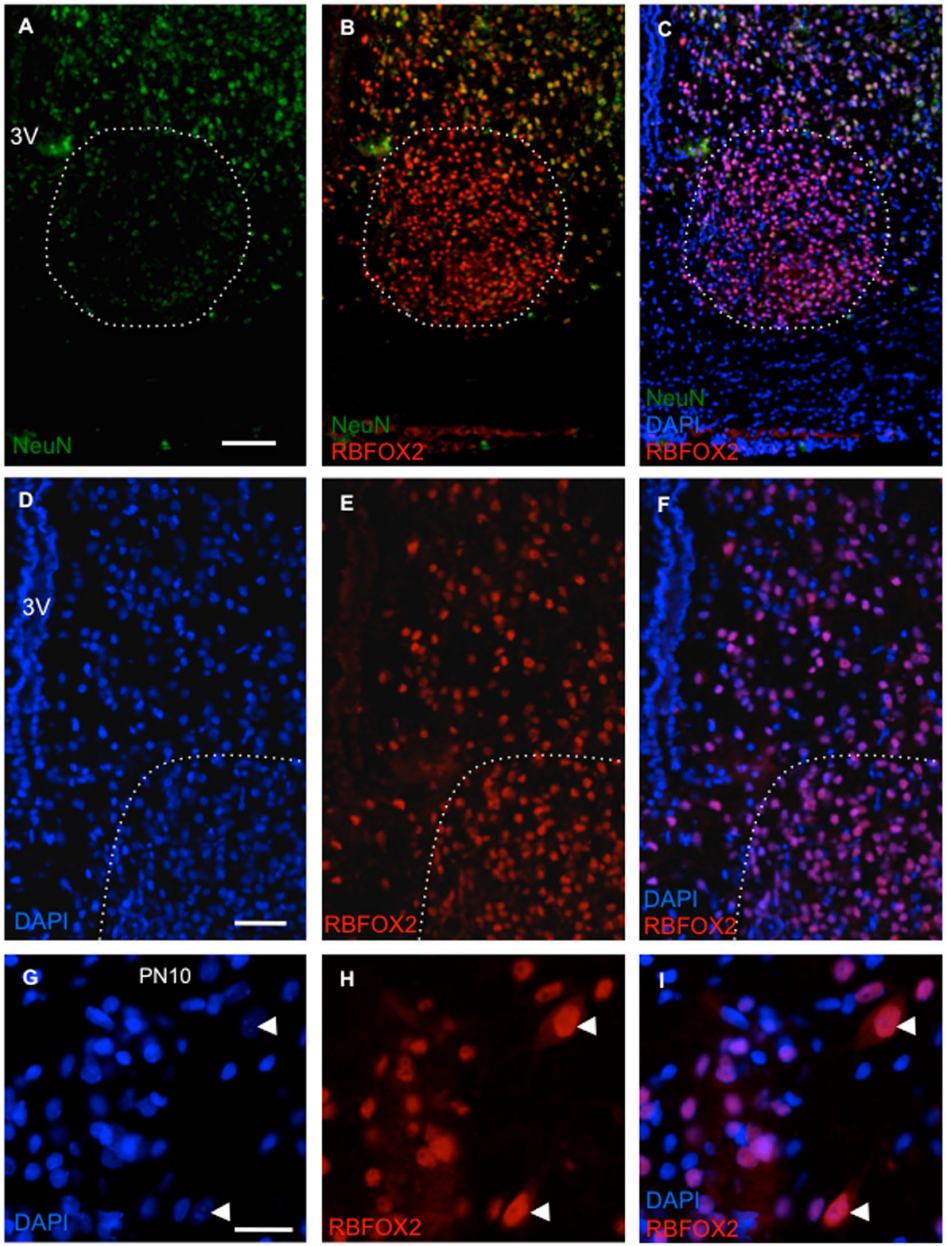
RBFOX2 is extensively expressed in rat suprachiasmatic nucleus (SCN). Representative fluorescence microscopic images of male PN50 and PN10 brain showing the distribution of RBFOX2 immunoreactivity in neurons. (**A**–**C**) Scarce and low-level expression of NeuN/RBFOX3 contrasted with abundant expression of RBFOX2 within the PN50 SCN (dashed line indicates the approximate confines of this brain region). Note the similar levels of RBFOX2 immunoreactivity in SCN and extra-SCN regions. (**D**–**F**) RBFOX2 is restricted to the nucleus of PN50 SCN neurons as indicated by co-localization with DAPI staining (Note invariant purple colour of co-localized cells imaged in (**F**)). (**G**–**I**) RBFOX2 is primarily restricted to the nucleus of PN10 hypothalamic neurons, but is also observed in the cytoplasm of isolated neurons. Arrowheads indicate two neurons exhibiting cytoplasmic expression of RBFOX2 (Note maintenance of red fluorescence when merged with DAPI image). Abbreviations: 3 V, third ventricle; DAPI, 4′,6-diamidino-2-phenylindole; NeuN, neuronal nuclei antigen/RBFOX3. Scale bars: A-C = 100 µm; D-F = 50 µm; G-I = 20 µm.

Given the identification of ‘MEKK’-containing Rbfox2 transcripts in rat cortex and SCN, we next used an antibody directed against this N-terminal epitope to analyse RBFOX2 protein expression in adult rat brain (Fig. 2). This analysis revealed abundant (MEKK) RBFOX2 protein in rat brain, including cortex (Figs 2 and S2), and also showed that this isoform ‘family’ (see results below) is similarly abundant in the SCN, a region where the related NeuN/RBFOX3 protein is unusually sparse (Fig. 2A–C). Microscopic inspection of RBFOX2 immunoreactivity within individual cells of the SCN did not reveal any distinction in RBFOX2 levels between NeuN-ve and the rare NeuN + ve cells (Fig. S2), and RBFOX2 was broadly expressed across the SCN with no apparent localization to sub-region (Fig. 2B). Further inspection of RBFOX2 immunoreactivity indicated an exclusively nuclear distribution in the adult rat brain regions analysed here (Fig. 2D–F). Expression of RBFOX2 in the nuclear region of neurons was confirmed by co-localization with MeCP2 (Fig. S3). In order to extend our understanding of the distribution of RBFOX protein in rat brain, further analysis of neonatal rat brain (Fig. 2G–I) revealed some neurons with distinctive RBFOX2 localization in extra-nuclear regions of the cells.

### Distinct populations of *Rbfox2* isoforms in the SCN and cortex

Following the initial identification of MEKK-containing transcripts in adult rat cortex (above), we cloned and sequenced a similar PCR product from adult rat SCN and were surprised to find a sequence with two internal variations (12 bp deletion at the start of exon 9, and a 40 bp full deletion of exon 10; a variant termed −12–40; Supplemental data SI1 and 2). Attempts to confirm this result revealed additional sequence variation, and so we undertook a systematic analysis of both cortex and SCN transcript sequences from multiple independent samples of rat brain (Table 1). This analysis revealed a diversity of transcripts in both brain regions, but at the same time, a clear distinction in isoform populations.

**Table 1:** Summary of Rbfox2 isoform populations in rat brain SCN and cortex.

Sequence variation(nt)	Rat Exon	Domain	Genbank identity	SCN clones	Cortex clones
None			Mm Isoform4	5	2
+32	*10*	CTD	Rno X 18	2	3
−12 + 32	9,*10*	CTD	Rno X 20	1	1
−3a-12	2,3,9	NTD&CTD	novel		2
−12–40	9,10	CTD	Mm X 27	12	4
−39	9	CTD	novel	1	
−93	5	RRM	novel		5
−93 + 73	5	RRM&CTD	Mm Isoform 9	1	
−93–12	5/9	RRM	novel		2
−93-12-40	5,9,10	RRM&CTD	novel		1
−3b-12-40	4,9,10	RRM&CTD	novel		2
Total Clones				**22**	**22**

Rbfox2 isoforms are identified by sequence variation (eg. + 32 = addition of 32 nucleotide sequence). Note two distinct –3 nt variations (3a and 3b). Six isoforms are novel, but others have Genbank annotations. Rno = rat; Mm = mouse. *10* = alternative exon 10.

In this sequence analysis of 44 clones (22 SCN, 22 cortex; each from 3 independent samples), a total of 6 loci of variation across the *Rbfox2* ORF were identified (Supplemental data SI1 and 2), with multiple different events at some of these sites, giving rise to a total of 11 distinct isoforms, including the ‘non-variant’ sequence identified above (Fig. 1C; Table 1, Supplemental data, SI1 and SI2). All of these isoforms are novel in this biological context, and therefore this study provides the first experimental evidence for the expression of these isoforms. It should be noted, however, that many of these isoforms are Gnomon-predicted from *ab initio* modelling (Table 1; www.ncbi.nlm.nih.gov/genome/annotation_euk/gnomon), and some involve documented alternatively spliced exons (see Discussion). ORF analysis indicates a direct correspondence between isoform sequence and our detected brain protein expression because all of the cloned isoforms maintain the N-terminal region recognized by the antibody used in the immunohistochemical analysis. Furthermore, our demonstration of (primarily) nuclear RBFOX2 in adult brain indicates the presence of functional nuclear localization signals (NLS) within the complement of different isoforms, and this appears to hold because, although many of the isoforms lack the known C-terminal NLS^13^ and the recently identified N-terminal NLS^13^ a conserved NLS (*KSTPKRLHVSNIPFRFRDPDLRQMFGQFGKI*) is identified in the majority of isoforms using the CNLA Mapper software. Remarkably, given the nature of the relatively short sequence insertions and deletions found here, only one of the sequenced variants is associated with premature termination of the ORF, and likely targeting for nonsense-mediated decay (NMD). This is the −93/+73 isoform, identical to a mouse isoform identified by Dredge & Jensen^25^ as targeted for NMD due to a nonsense codon more than 50–55 bp upstream of an exon-exon junction. All other isoforms exhibit maintenance of a similarly sized ORF, terminating in either ‘FAPY’ or ‘TEVT’. This organization is dependent upon the use of different (alternative frame) termination codons that are spaced 7 bp apart at the 3′ end of the coding sequence.

Clearly, the pattern of differential isoform expression between SCN and cortex is complex and does not have an overt functional distinction, particularly given that the functional activities of each isoform have not been experimentally defined. However, our data reveal a 3-fold higher abundance of the −12–40 isoform in SCN compared with cortex (Table 1), and this isoform also represents >50% of the SCN isoforms. The −12 bp deletion results in a loss of the amino acid sequence SLPLV from the N-terminal region of the CTD, and the −40 deletion results in an almost complete loss of sequence identity from VYQD in the CTD until the end of the coding sequence (Supplemental data, SI4 and SI5). Given the differential expression of this isoform, we therefore selected the −12–40 isoform for functional analysis (see below; Genbank accession number: MF177287).

### RBFOX2 target sequences rat brain isoforms

Although multiple factors contribute to differential splicing of neuronal genes^4^, RBFOX protein auto-regulation may be involved, and therefore an analysis of rat genomic sequence at the Rbfox2 locus was conducted in order to identify patterns of RBFOX binding sites ([U]GCAUG) that would be consistent with auto-regulation. This analysis (see Fig. 1C) provided evidence for Rbfox-mediated regulation of Rbfox2 isoform generation. Of note, there are consensus sites proximal to exon 10 and the alternative exon 10 which would be consistent with exon exclusion and inclusion respectively, and also proximal sites around the alternatively utilized exon 5. In other cases, such as the 12 bp loss from exon 9, no consensus sites are found, indicating alternative mechanisms of sequence variation in *Rbfox2* isoforms.

### RBFOX splicing targets in SCN genes

**Figure 3:**
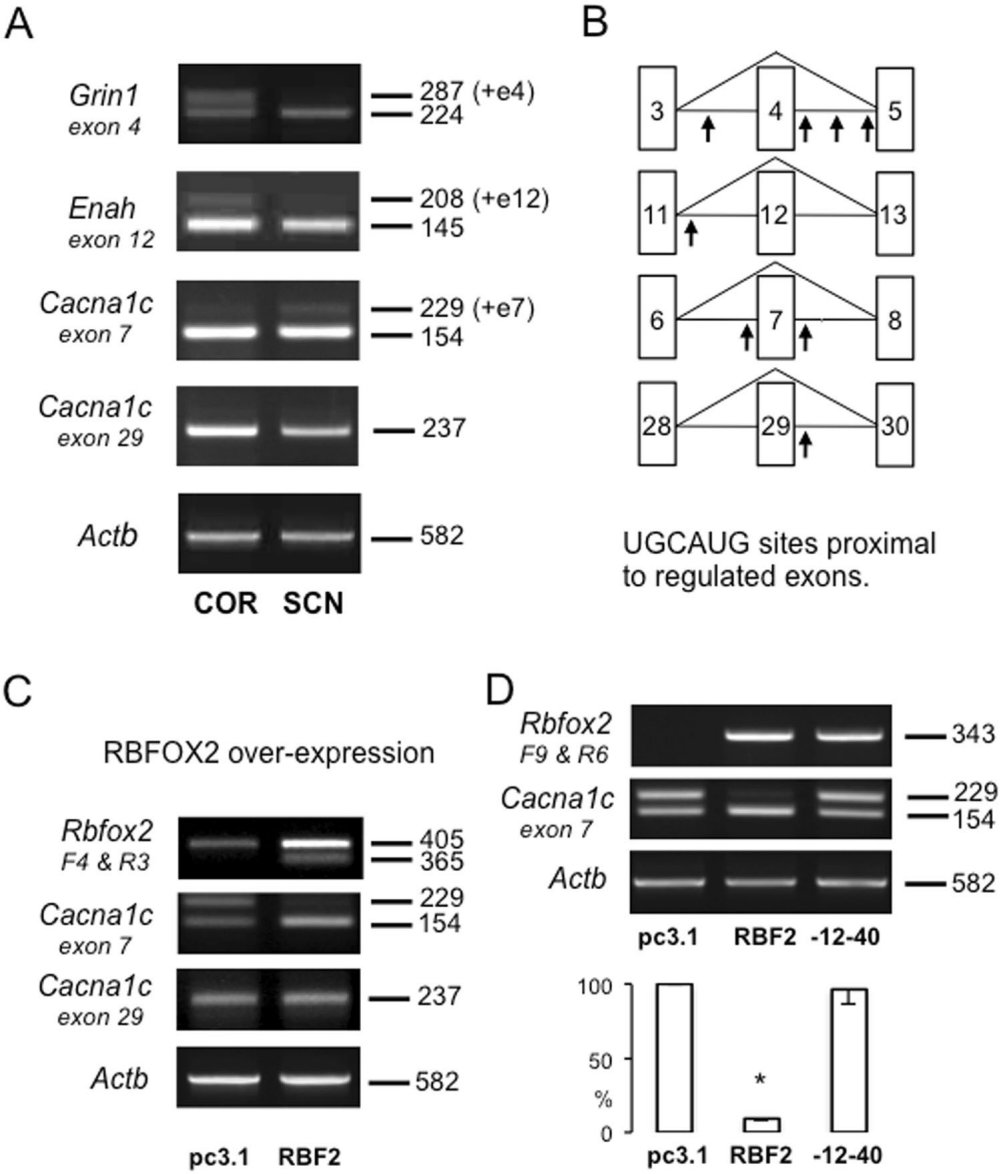
Differential expression of RBFOX-target exons in rat brain cortex (COR) and suprachiasmatic nucleus (SCN), and differential activity of RBFOX2 isoforms. (**A**) Representative agarose gel electrophoresis images of RT-PCR analysis using primers directed at transcript regions spanning exons that are documented as either included (*Grin1* exon 4, *Enah* exon 12, or excluded (*Cacna1c* exons 7 & 29) by RBFOX protein activity. Exons are numbered according to currently annotated rat gene transcripts (see text). Numbers on right indicate amplicon sizes in bp. (**B**) Schematic representation of gene exon structure. Arrows indicate position of proximal (within 500 nt) UGCAUG (consensus RBFOX-binding) sequences. (**C**) Representative agarose gel electrophoresis images of RT-PCR analysis of PC12 cell RNA showing the effect of RBFOX2 (RBF2) over-expression (vs. emply vector, pc3.1) on exon usage in target genes. Note the marked reduction in *Cacna1c* exon 7 inclusion in the RBF2 lane, but no effect on *Cacna1c* exon 7. (**D**) Comparative analysis of non-variant RBFOX2 (RBF2) and −12–40 RBFOX2 variant (−12–40) over-expression on *Cacna1c* exon 7 exclusion in PC12 cells. Upper: Representative agarose gel electrophoresis images of RT-PCR analysis. Note the Rbfox2 primers are specific for exogenous Rbfox2 transcripts. Lower: Quantitative comparison of multiple experiments showing a significant reduction of exon 7 inclusion with RBF2 (*p < 0.05 [p < 0.000, 2-tailed] Independent Student’s t test) but no significant effect of the −12–40 variant (p > 0.05 [p = 0.743, 2-tailed] Independent Student’s t test. Levene’s test for equality of variance: RBF2: F = 6.179, p = 0.047; −12–40: F = 7.762, p = 0.032. Values are Mean ± SEM; n = 4 replicate transfections in each group. Note that gel images have been cropped for clarity; full-length gel images for each amplicon are shown in Fig. S4.

Previous studies of RBFOX protein function have identified multiple exons that are included/excluded from target gene transcripts through RBFOX-dependent mechanisms (see Discussion). In order to investigate comparative RBFOX activity in the SCN, four of these exon targets were analysed using RT-PCR to provide a comparison of exon inclusion/exclusion in the SCN and cortex (Figs 3A and B; S4). Initially, we analysed exon 4 (Transcript: ENSRNOT00000037725.5), of the NMDA receptor gene, *Grin1*, which in addition to being a known RBFOX target exon^9, 23^ [termed exon 5^9^], is also known to be differentially regulated in SCN (exon excluded) compared with cortex (exon included)^16, 17^. Consequently, our demonstration that this exon is excluded from SCN (but not cortex) transcripts in our samples (Fig. 3A), is not only indicative of a region-specific difference in RBFOX activity, but also serves to confirm SCN sample integrity, given the previous *in situ* hybridization data for SCN expression^16, 17^.

We have also demonstrated brain region-specific regulation in two other genes that contain known RBFOX target exons, but which have not previously been analysed in the SCN (Fig. 3A). First, *Enah* (aka *Mena*) exon 12 (Transcript: ENSRNOT 00000085188.1), which has a lower expression in the SCN, consistent with a loss of RBFOX activity^7, 26^. Second, *Cacna1c* (aka *Cav1*.*2*) exon 7 (Transcript: ENSRNOT00000052017.6) that is clearly expressed in SCN, but only minimally in cortex (Fig. 3), again, consistent with a loss of RBFOX activity^10^ (termed exon 9*). A quantitative analysis of Cacna1c exon 7 inclusion revealed a significantly higher level of exon 7 included transcripts in SCN when expressed as a percentage of total PCR product (SCN: 7.0 ± 0.4%, Cortex: 1.7 ± 0.4%; p < 0.05, Independent Student’s t test, n = 3/group, values are Mean ± SEM). In contrast, *Cacna1c* exon 29 inclusion is complete in both SCN and cortex, which is not surprising given that regulated exclusion of this exon has been shown to terminate on embryonic day 16 in mouse brain^10^ (termed exon 33). Overall, these analyses are consistent with a relatively lower level of RBFOX spicing activity in SCN compared with cerebral cortex.

### Distinct population of Rbfox2 isoforms in a neuronal cell line

To establish a cellular model for the investigation of *Rbfox2* isoform function, rat PC12 pheochromocytoma cells were cultured and analysed for expression of both endogenous *Rbfox2*, and known RBFOX2 target genes. RT-PCR with the RBFF6 and RBFR3 primers used for rat brain analysis produced abundant expression of a product that appeared similar to the PCR bands obtained with SCN and cortex RNA (Fig. 1A). However, cloning and sequencing of these cDNAs revealed a set of sequences largely distinct form either of the rat brain samples (Supplemental data, SI3). Of 6 sequenced clones, one was identical to the original ‘non-variant’ sequence cloned from rat cortex, but the remaining 5 clones (−12 × 2; −3b-12; −2–40 + 43 × 2) were distinct from both the SCN and cortex populations, with a notable absence of both the −12–40 isoform, and any −93 isoforms in the PC12 population. In contrast, an isoform containing a substitution of exon 10 (−40) with a well-established alternative exon 10* (+43)^7^ was found. This result is indicative of the undifferentiated state of the PC12 cells because the alternative (+43) exon is known to be absent in brain mRNAs^7^. However, this clone also contained a novel −2 bp deletion, an apparent micro-exon flanked by GT and AG sequences. Overall, the 3 variations in this clone result in a truncated predicted protein sequence (Supplemental data, SI3).

Initial analysis of RBFOX target genes in PC12 cells revealed abundant expression of both exon 7-included and exon 7-excluded *Cacna1c* transcripts (Fig. 3C and D), making this exon an ideal target for functional analysis of Rbfox2 isoforms.

### The SCN-abundant RBFOX2 isoform is functionally impaired

Given that the (−12–40) isoform is the most abundant clone in the rat brain sample set, and furthermore distinguishes the SCN and cortex populations, a functional analysis of this isoform was conducted using the equivalent (‘non-variant’) isoform for comparison. This analysis focused on two aspects of RBFOX function using PC12 cells as an experimental model: (i) effect on inclusion of *Cacna1c* exon 7 (see above), and (ii) effect on gene expression (selecting *Egr1* as a documented example gene)^27^.

The non-variant Rbfox2 construct was functionally verified in initial transfection experiments that demonstrated a marked decrease in *Cacna1c* exon 7 inclusion following 24 h of over-expression (Fig. 3C). In contrast, no change in expression of *Cacna1c* exon 29 was observed (Fig. 3C). Interestingly, analysis of *Rbfox2* expression using primers RBFF4 and RBFR3, that detect both endogenous and exogenous *Rbfox2* transcripts, revealed not only an anticipated over-expression of *Rbfox2*, but also (sequence-verified) expression of a (‘−40’; 365 bp) product in the *Rbfox2*-transfected cells, presumably reflecting exclusion of the 40 bp exon 10 from endogenous *Rbfox2* transcripts. Following this verification of functional RBFOX2 activity in the transfection paradigm, additional experiments were conducted to quantitatively compare this activity with the −12–40 isoform. Despite similar levels of construct expression (verified by amplification of a PCR product derived from the 5′ UTR region of pcDNA3.1 coupled with a Rbfox2-specific reverse primer; Fig. 3D), expression of the −12–40 isoform did not significantly modify *Cacna1c* exon 7 inclusion (Fig. 3D).

In contrast to the differential splicing activity of the rat brain Rbfox2 constructs demonstrated above, neither construct significantly modified *Egr1* mRNA levels in this cellular model. Fold changes in *Egr1* mRNA level for the different transfections compared to pcDNA3.1 were: ‘non-variant’ Rbfox2, 0.92 ± 0.19, and −12–40, 1.12 ± 0.17 (n = 4 replicate transfections in each group; ANOVA: F = 0.465 (11), p = 0.642). *Actb* is also a documented RBFOX target^23^ and although not quantitatively analysed here, expression did not appear to be modified by rat brain Rbfox2 construct over-expression (Fig. 3C and D).

## Discussion

The RBFOX family of RNA binding factors are established regulators of neuronal gene expression^4^. Individual family members are known to be required for aspects of adult brain function^28^, but, in general, the roles of these RBPs in adult brain are largely undefined. In the present study, we have shown that *Rbfox2* is expressed in a remarkable diversity of isoforms in the adult brain, and also that different brain regions have characteristic isoform populations. This analysis has also revealed that RBFOX activity in the RBFOX3/NeuN-depleted SCN, appears to be compensated by a change in *Rbfox2* isoform population rather than a change in expression levels. In the SCN, an abundance of a CTD-variant *Rbfox2* isoform (−12–40) is associated with distinct patterns of exon utilization in RBFOX target genes. Previous studies have highlighted individual RBFOX isoforms in the brain, for example a cytoplasmic variant of RBFOX1^29^, but the diversity of neuronal isoform variation has not previously been documented. Our comparative analysis of the cerebral cortex with the SCN has perhaps contrasted two extremes, given the unusual, RBFOX3-negative nature of the majority of SCN neurons, and so further analysis is required to extend these findings to other neuronal populations in order to identify the extent of variation across the brain.

Our study has also demonstrated a functional correlate of the distinct *Rbfox2* isoform populations, in that the SCN-enriched CTD-variant *Rbfox2* isoform lacks the functional activity of an equivalent non-variant isoform. As a consequence, the relative loss of RBFOX2-mediated exon exclusion from a L-type calcium channel gene, *Cacna1c*, may underlie the presence of this exon within SCN *Cacna1c* transcripts. These findings have additional relevance to this study because inclusion of this exon is known to be developmentally down-regulated in brain cortex^10^, providing further evidence of an immature molecular phenotype in the SCN^14, 19^. Our control experiments have confirmed RBFOX2-mediated exclusion *Cacna1c* exon 7, as originally demonstrated by Tang and colleagues^10^, and provide an interesting *in vivo* context for this activity because these authors showed that the *Cacna1c* exon responds to relatively small changes in RBFOX2 levels. Hence, differences in the relative proportions of *Rbfox2* isoforms between SCN (eg. −12–40, >50%) and cortex (<20%) are likely to be functionally relevant.

The major importance of alternative splice regulation in neurons lies in the generation of protein isoforms with altered properties; variant ion channels, for example, which affect neurophysiological output, and therefore information processing^30^. Inclusion of *Cacna1c* exon 7 has been shown to modify the electrophysiological properties of the *Cacna1c*-encoded CaV1.2 channels^10^. Regulated splicing of the CaV1.2 channel is interesting in the context of this study because CaV1.2 is involved in resetting of the circadian clock within the SCN^31^. However, the *Cacna1c* gene is subject to extensive splice variation, and the physiological significance of much of this variation remains undefined^32^.

We have also provided evidence of other RBFOX-targeted gene exons, *Grin1* exon 4^5^ and *Enah* exon 12^7, 26^ that are differentially expressed in the SCN, also reflecting an apparent relative loss of RBFOX activity. For the NMDA receptor subunit gene *Grin1*, we have confirmed an absence of the variable exon 4 in SCN mRNAs^16, 17^ and, conversely, clear exon 4 expression in cerebral cortex^33, 34^. Inclusion of *Grin1* exon 4 is directly mediated by RBFOX proteins, presumably via a cluster of binding sites downstream of exon 4^5^. For the *Enah* gene, not previously been studied in the SCN, we show for the first time that exon 12, a known RBFOX-regulated exon^26^ is included in cortical transcripts, but not in SCN transcripts.

Exons in the *Cacna1c*, *Grin1* and *Enah* genes are experimentally verified RBFOX2 targets as established in an extensive HITS-CLIP analysis of individual RBFOX proteins^23^. In the latter study, genome-wide RBFOX exon targets are ranked according to FDR score: *Rbfox2* (+93) is ranked 9, *Grin1* (exon5) ranked 4, and *Cacna1c* (exon 9) ranked 554. Also, in this study, the potassium channel gene *Kcma1* is identified as a major target of RBFOX with exons ranked in positions 198, 376 and 669. We have not studied the *Kcnma1* in the current study but previous work has identified that the ‘STREX’ splicing site^18^ lacks inserts in the SCN, again consistent with relative loss of RBFOX activity in these neurons due to prediction of exon inclusion in the HITS-CLIP analysis^18^.

The generation of *Rbfox2* isoforms observed in the current study may be multifactorial, involving different RBPs, and other molecular mechanisms. However, given the extensive evidence of *Rbfox* autoregulation^35^, it is likely that differential isoform generation is at least partly RBFOX-regulated. For example, the abundance of the −93 isoform in cortex relative to SCN would be consistent with RBFOX3/NeuN enhanced skipping of RRM exons that generate this dominant negative isoform^25, 36^. In contrast, the relative absence of RBFOX3/NeuN in the SCN removes this influence on *Rbfox2* isoform generation^25^, resulting in a distinct population of neuronal isoforms. Our analysis of genomic sequence around regulated *Rbfox2* exons has identified potential target sequences that are proximally positioned to mediate auto-regulation. This analysis is supported by recent genome-wide analyses of RBFOX target sites^7, 23^ that identify the ‘40’ and ‘93’ exons as targets. Many RBFOX consensus sites are located distally (>500 nt) from exons, but it should be noted that RNA bridges can mediate distal regulation^37^.

Our major finding of a multiplicity of *Rbfox2* isoforms in the adult brain raises the question of protein function associated with these individual variations. Our sequence evidence implies that these variants are translated because most sequence variations are integrated with alternative stop codon usage that maintains an open reading frame. Previous evidence also shows that alternative *Rbfox* transcripts are associated with translated proteins and not simply subject to NMD^5^. Consequently, it is possible to generate many different proteins with different functional properties, relating to one or more aspects of RNA binding, protein interaction and cellular (nuclear/cytoplasmic) localization. Previous studies have identified RBFOX2 isoforms with dominant negative activity, for example the −93 isoform can act as a dominant negative because the RRM is partially deleted leading to reduced RNA binding but maintained association with other splicing factors^36^. Our results may indicate a requirement for such activity in cortical neurons where these isoforms are more abundant. The relatively lower level of these isoforms in SCN would be consistent with a need to compensate for the loss of RBFOX3/NeuN in these neurons. However, our current analysis of Rbfox2 expression in the SCN is generally consistent with a relative loss of RBFOX activity that appears to be appropriate for the required splicing patterns in functional SCN genes.

The SCN is characterized by an abundance of the −12–40 isoform that codes for a CTD-variant isoform. Alternative usage of the 40 bp exon 10 in mouse brain and muscle has been documented previously^38^, but the 40 nt exon 10 was substituted with a 43 nt exon rather than deleted. In the rat brain areas analysed here, we have found that deletion of the 40 bp exon 10 is exclusively associated with an additional, novel deletion of an upstream 12 bp sequence. The resultant amino acid changes in the CTD are significant, involving loss of alanines, (33 A’s in the ‘non-variant’ protein compared with 16 in −12–40, and loss of a ATATAATAAAAAAAAY sequence) and addition of glutamine (+5) and proline (+11) residues, resulting in five closely-spaced QP dimers. These changes may be crucial for splicing function because the CTD is required for RBFOX2 splicing activity^39–42^, acting, at least partly, to mediate protein interactions^12, 41^. CTD variation may relate to the recently demonstrated, but unexplained selection of distinct RBFOX binding sites by RBFOX2^8^. Alternatively, the variant CTD may confer other, non-splicing, functions related to mRNA processing (see below), possibly explaining the enrichment of glutamines^43^ and QP repeats^44^.

Although much of the work on RBFOX proteins has necessarily focused on their role in splicing, recent work has provided evidence for other regulatory roles. Both RBFOX1^45^ and RBFOX2^46, 47^ can modify mRNA levels in the cytoplasm via actions at the 3′ UTR. In the adult rat brain including the SCN, we found no evidence of cytoplasmic RBFOX2. However, other recent work has shown that RBFOX2 can have a more general role in gene regulation, acting (largely) as a transcriptional repressor in conjunction with polycomb complex 2 (PRC2)^27^. The RBFOX2 protein domain interacting with PRC2 is the CTD, shown here to be significantly modified in the SCN-abundant −12–40 isoform. A consequent change in gene repression in the SCN may therefore explain some of the unusual patterns of gene expression in SCN neurons^14, 19^. In the current study, one of the RBFOX2-suppressed genes, *Egr1* ^27^, was measured in an RBFOX2 over-expression paradigm, but no consistent changes in *Egr1* expression were observed. Further studies, possibly in other cellular contexts^27^, are required to confirm these regulatory actions of RBFOX2.

In conclusion, our demonstration of distinct populations of *Rbfox2* variants in the adult brain is consistent with our widening knowledge of neuronal transcript diversity, as demonstrated in current single-cell sequencing projects^48^. Our additional finding of further (novel) variants in PC12 cells is suggestive of a multiplicity of *Rbfox2* transcript populations that may be a defining feature of individual cell types. In the SCN, where RBFOX3/NeuN is largely absent, our finding that there are similar levels of *Rbfox2* transcripts and protein but distinct isoform populations is indicative of a novel cellular strategy for directing cell-specific RBFOX activity. Further studies are required to understand how the changes in *Rbfox* expression may relate to specific SCN function; for example, ‘immature’ aspects of SCN physiology^49^, or possibly the requirement for daily cycles of regulated splicing^18, 50, 51^.These studies must take into account other RBPs such as NOVA, which can act synergistically with RBFOX proteins to regulate neuronal gene splicing^52^, and also activity of RBFOX1. At a more general level, the current insights into brain *Rbfox2* gene expression may have clinical relevance because the RBFOX-regulatory network is associated with autism, and RBFOX-target genes, including *Cacna1c*, are recognized autism susceptibility genes^23^.

## Methods

### Animals

Sprague-Dawley male rats were used in accordance with both the UK Animals (Scientific Procedures) 1986 Act of Parliament, and Cardiff University ethical review. All experimental protocols were approved by, and conducted under, UK Home Office licencing. The health status of the animals was monitored by a veterinarian. Rats were maintained in standard laboratory conditions (14:10 light:dark cycle, lights on: 05.00 h) with *ad libitum* access to rodent food and drinking water, and sampled at 17.00 h. For immunohistochemical analysis of postnatal day 50 (PND50) brain, animals were terminally anaesthetized with sodium pentobarbitone (150 mg/kg, i.p), and perfused via the ascending aorta with phosphate buffered saline (PBS), followed by 4% paraformaldehyde in 0.1 M phosphate buffer (PFA). Dissected brains were then post-fixed (PFA, overnight, 4 °C). Alternatively, for PND10 rats killed by a Schedule 1 method, brains were rapidly dissected prior to fixation in 4% PFA (24 h, 4 °C). Both PND50 and PND10 brains were then cryoprotected (20% sucrose in 0.1 M phosphate buffer; PND50: overnight, 4 °C; PN10: 24 h, 4 °C), and stored briefly at –80 °C prior to sectioning. For RT-PCR analysis of PND50 brain, rats were killed by a Schedule 1 method, and discrete punches of SCN and cerebral cortex (parietal) tissue were sampled using a method^53^ optimized by the use of a rat brain matrix (RBMA-300C, World Precision Instruments Inc., Sarasota, FL, USA) and blunted 15 G needles. Tissue from 3 rats was pooled in each sample used for RT-PCR.

### Immunohistochemistry

Coronal sections of rat brain (12 µm; bregma 0.0 mm to −2.0 mm)^54^ were prepared using a cryostat (Leica CM1900; Leica Imaging Solutions Ltd., Cambridge, UK), and mounted on glass slides (SuperFrost Plus, VWR International Ltd., East Grinstead, West Sussex, UK). Following brief drying, slides were stored at −80 °C prior to analysis. Sections containing the SCN were selected, and protein expression was analysed using fluorescence immunohistochemistry^55^. The primary antibodies used for immunohistochemistry (RBFOX2, RBM9 IHC-00199, Bethyl Laboratories Inc., Montgomery, TX, USA; NeuN/RBFOX3, MAB377, Millipore, Temecula, CA, USA), and MeCP2 (4B4, Active Motif, Carlsbad, CA, USA), have been used in our previous studies^19, 55, 56^, and have been validated both positively (excitation light-specific detection), and negatively (absence of detection when the primary antibody is omitted). The epitope recognized by IHC-00199 maps to a region in the N-terminal domain (residues 1–50 of hRBFOX2; NP_055124.1) that is 100% conserved between human and rat. Note that this antibody will not distinguish RRM and CTD variants of RBFOX2. The secondary antisera used were: Cy3-conjugated sheep anti-rabbit IgG (Sigma Aldrich, Dorset, UK), and Alexa Fluor 488-conjugated donkey anti-mouse IgG, (Molecular Probes Inc, Eugene, OR, USA). Following washing, sections were mounted in Vectashield with DAPI (Vector Laboratories, Burlingame, CA, USA), stored at 4 °C, and then viewed with a fluorescence microscope (Leica DM-LB, Leica). Representative images were photographed with a Leica DFC-300FX digital camera linked to Leica QWin software (V3), and assembled in Photoshop (CS2, Adobe Systems Inc., San Jose, CA, USA). Expression of RBFOX2 in the SCN was confirmed in at least 6 brain sections from 2 rats.

### RT-PCR analysis of rat brain mRNAs

Total cellular RNA was extracted from rat brain samples using Trizol (Invitrogen protocol, Thermo Fisher Scientific, Waltham, MA, USA) and DNaseI-purified (Promega protocol; Promega, Madison, WI, USA). cDNA was generated with the Superscript II protocol (Life Technologies, Thermo Fisher Scientific) using an Oligo (dT) primer. PCR was conducted using standard procedures with either REDTaq ReadyMix (Sigma, Aldridge, St.Louis, MO, USA), or Q5 Hot-Start High-Fidelity DNA polymerase (NEB, Ipswich, MA, USA). Oligonucleotides used for amplification are listed in Table S1. Amplified products were visualized after agarose gel electrophoresis, with reference to a DNA ladder (Hyperladder I, Bioline, London, UK or 1 kb ladder, Promega), using GeneSnap (Syngene, Frederick, MD, USA), and relative levels of PCR bands measured using Image J^57^. For cloning and sequence analysis, PCR products were purified (Qiaex II gel extraction kit, Qiagen, Hilden, Germany) and either directly ligated into pGEM-T (Promega protocol), or, in the case of Q5-amplified products, ‘A-tailed’ (Promega protocol) prior to ligation. Ligations were transformed into JM109 cells (Promega), and transformants were selected for plasmid purification (Wizard SV Miniprep protocol, Promega). PCR products were then sequenced (Eurofins MWG Operon, Ebersberg, Germany).

For the analysis of Rbfox2 isoform distribution within different brain areas, a total of 44 clones were sequenced (22 SCN comprising 10, 6 and 6 clones from SCN sample pools (3 rats in each pool) A, B & C respectively; 22 cortex comprising 10, 6 and 6 clones from Cortex sample pools (3 rats in each pool) A, B & C respectively). For the analysis of RBFOX splicing targets in SCN and cortex, exon exclusion/inclusion was verified in at least two independent PCR reactions using independent brain samples from the same sample pools used for Rbfox2 isoform analysis. A quantitative analysis of *Cacna1c* exon 7 inclusion in SCN and cortex was conducted by measuring the relative levels of exon 7 included, and excluded products (see Results).

### Quantitative RT-PCR analysis

For the quantitative analysis of *Egr1* mRNA levels, real-time PCR analysis was performed with the qPCRBIO SyGreen Mix (PCR Biosystems protocol, PCR Biosystems Ltd, London, UK) on a Mx3000P machine (Agilent, Santa Clara, CA, USA) using the 2^−ΔΔ*CT*^ method for quantitation. For this analysis, reverse transcription reactions (see above) were conducted using a mixture of oligo (dT) and an 18S-specific primer in order that 18S RNA (template diluted 1/1000 for QPCR) could be used for reference^58^. This approach for normalization was adopted due to the reported widespread effects of RBFOX2 across the transcriptome^27^.

### Expression of Rbfox2 isoforms in cell culture

PC12 cells (Gift of Prof. D. Murphy, University of Bristol, UK) were grown in DMEM with 10% horse serum, 5% Fetal Bovine Serum and 1x antibiotic/antimycotic (Invitrogen) at 37 °C and 5% CO_2_. For experimental analysis, cells (1 × 10^5^/well of a 12-well plate;) were transfected (TransFast protocol, Promega) with Rbfox2 constructs (250 ng, PureYield plasmid, Promega protocol) and maintained for 24 h prior to RNA extraction using Trizol (Invitrogen protocol). RNA samples were reverse transcribed as described above and transcripts were analysed using end-point PCR (REDTaq Readymix) with cycle numbers appropriate for individual transcripts (range: 28–34 cycles). Following agarose gel electrophoresis, gel images were captured (GeneSnap, Syngene) and relative levels of PCR bands measured using Image J^57^. Rbfox2 expression constructs were based on isoform sequences obtained in the analysis of rat brain transcripts. Selected Rbfox2 sequences were re-amplified using primers containing 5′ *HindIII* or *BamHI* sites, sequence-verified, and then ligated into the MCS of pcDNA3.1(+). In order to optimize expression of RBFOX2 protein, the 5′ UTR was modified with a single (non-coding) base change to generate a consensus Kozak sequence. For the analysis of endogenous PC12 Rbfox2 transcripts, PC12 cells were similarly plated and maintained in the absence of transfection. Following RNA extraction, PC12 Rbfox2 transcripts were cloned and sequenced as described above (total of 6 *Rbfox2* clones).

### Bioinformatics and statistical analysis

Genomic and transcript analysis was performed using the UCSC and Ensembl Genome Browsers (genome.ucsc.edu; ensembl.org). Sequence identity was initially confirmed using BLAST (blast.ncbi.nlm.nih.gov), and sequence comparisons were conducted with Clustal Omega (ebi.ac.uk/Tools/msa/clustalo). Nuclear localization signals were identified using CNLS MAPPER (nls-mapper.iab.keio.ac.jp/cgi-bin/NLS_Mapper_form.cgi). Statistical analysis was conducted with IBM SPSS Statistics version 20 (IBM, New York, USA) by applying different tests as indicated in Results, and Levene’s test for equality of variance. The requirement to use multiple end-point PCR reactions for comparative analysis of endogenous splice variation across single exons necessarily compromises inter-assay variation, and therefore independent Student’s t-tests were used to compare the activity of the two expression constructs with control transfections.

## Electronic supplementary material

Supplementary information
