# Supplementary material for: Novel Rbfox2 isoforms associated with alternative exon usage in rat cortex and suprachiasmatic nucleus
Source: Sci Rep. 2017 Aug 30;7:9929. doi: 10.1038/s41598-017-10535-3 (PMC5577181; doi:10.1038/s41598-017-10535-3)
Supplement: Supplementary file 1 — Supplementary information [file 41598_2017_10535_MOESM1_ESM.pdf]

Novel *Rbfox2* isoforms associated with alternative exon usage in rat cortex and suprachiasmatic nucleus.

L.M.M. Partridge

D.A. Carter

Supplemental Information.

SI1. Rat brain Rbfox2 cDNA: Exon structure, domain boundaries, and sites of variation.

ATGGAGAAAAAGAAAATGGTAACTCAGGGTAACCAGGAGCCAACAACAACTCCTGACGCAATGGTTCAG
CCTTTTACTACCATCCCATTCCCACCACCTCCACAGAACGGAATTCCCACAGAATATGGAGTGCCACAC
ACTCAGGACTATGCCGGCCAGACCAGTGAACATAACCTGACACTCTACGGGAGTTCACAGCCCCATGGA
GAACAGAGTAGCAATTCACCCAGCAACCAGAATGGATCTCTCAC**GCA**GACAGAAGGTGGAGCACAGACA
GACGGACAACAGTCACAGACACAAAGTAGTGAAAATTCAGAGAGTAAATCCACGCCCAAG**CGA**CTACAT
GTCTCTAATATTCCCTTCCGCTTCCGGGACCCTGACCTCCGGCAGATGTTTGGG**CAG**TTTGGCAAAATC
CTAGATGTGGAAATAATCTTTAATGAGCGCGGTTCCAAG**GG**ATTCGGGTTCGTAACTTTCGAGAATAGT
GCTGATGCAGACAGGGCCAGGGAGAAATTGCACGGCACCGTGGTAGAGGGCCGTAAAATCGAGGTG**AAT**
AATGCTACCGCACGGGTCATGACCAACAAGAAGATGGTCACACCATATGCAAATGGCTGGAAGTTAAGC
CCAGTAGTTGGAGCTGTGTACGGCCCTGAGTTATATGCAGCATCCAGCTTTCAAGCTGATGTGTCCCTA
GGCAATGAGGCGGCTGTGCCCTTGTCA**GGA**AGAGGGGGCATCAACACTTACATTCCTCTAATCA**GTCTC**
CCTTTAGTTCCTGGCTTCCCTTACCCAACTGCAGCCACCACAGCAGCCGCTTTCAGAGGAGCCCATCTG
AGGGGCAGAGGGCGGACAGTGTATGGTGCAGTGCGAGCGGTACCTCCAACAGCCATCCCCGCCTATCCA
GG**TGTGGTTTACCAGGACGGATTTTACGGTGCTGACCTCTATG**GTGGATATGCAGCCTACAGATATGCA
CAGCCTGCTACTGCAACCGCAGCCACAGCTGCTGCAGCCGCTGCAGCCGCTTACAGCGACGGTTACGGC
AGGGTGTACACAGCTGACCCCTACCATGCCCTCGCCCCTGCCGCCAGCTATGGAGTTGGCGCTGTGGCG
AGTTTGTACCGAGGTGGCTACAGCCGATTTGCCCCCTACTGAAGTGACGTGAGAC

Exons are alternately black and blue font.

GCA -3a
GTCTCCCTTTAG -12/-39
TGTGGTTTACCAGGACGGATTTTACGGTGCTGACCTCTAT -40
CAG -3b
G +32
GG -93/+73
CGA **AAT** start and end of RRM
GGA start of CTD

SI2. *De novo* sequenced nucleotide, and predicted amino acid sequences of rat brain Rbfox2 isoforms.

'Minor' sequence variations (vs. Genbank) are highlighted in purple. These may represent polymorphisms or technical variants (PCR/sequencing error).

Non-variant

ATGGAGAAAAAGAAAATGGTAACTCAGGGTAACCAGGAGCCAACAACAACTCCTGACGCAATGGTTCAG
CCTTTTACTACCATCCCATTCCCACCACCTCCACAGAACGGAATTCCCACAGAATATGGAGTGCCACAC
ACTCAGGACTATGCCGGCCAGACCAGTGAACATAACCTGACACTCTACGGGAGTTCACAGCCCCATGGA
GAACAGAGTAGCAATTCACCCAGCAACCAGAATGGATCTCTCACGCAGACAGAAGGTGGAGCACAGACA
GACGGACAACAGTCACAGACACAAAGTAGTGAAAATTCAGAGAGTAAATCCACGCCCAAGCGACTACAT
GTCTCTAATATTCCCTTCCGCTTCCGGGACCCTGACCTCCGGCAGATGTTTGGGCAGTTTGGCAAAATC
CTAGATGTGGAAATAATCTTTAATGAGCGCGGTTCCAAGGGATTCGGGTTCGTAACTTTCGAGAATAGT
GCTGATGCAGACAGGGCCAGGGAGAAATTGCACGGCACCGTGGTAGAGGGCCGTAAAATCGAGGTGAAT
AATGCTACCGCACGGGTCATGACCAACAAGAAGATGGTCACACCATATGCAAATGGCTGGAAGTTAAGC
CCAGTAGTTGGAGCTGTGTACGGCCCTGAGTTATATGCAGCATCCAGCTTTCAAGCTGATGTGTCCCTA
GGCAATGAGGCGGCTGTGCCCTTGTCAGGAAGAGGGGGCATCAACACTTACATTCCTCTAATCAGTCTC
CCTTTAGTTCCTGGCTTCCCTTACCCAACTGCAGCCACCACAGCAGCCGCTTTCAGAGGAGCCCATCTG
AGGGGCAGAGGGCGGACAGTGTATGGTGCAGTGCGAGCGGTACCTCCAACAGCCATCCCCGCCTATCCA
GGTGTGGTTTACCAGGACGGATTTTACGGTGCTGACCTCTATGGTGGATATGCAGCCTACAGATATGCA
CAGCCTGCTACTGCAACCGCAGCCACAGCTGCTGCAGCCGCTGCAGCCGCTTACAGCGACGGTTACGGC
AGGGTGTACACAGCTGACCCCTACCATGCCCTCGCCCCTGCCGCCAGCTATGGAGTTGGCGCTGTGGCG
AGTTTGTACCGAGGTGGCTACAGCCGATTTGCCCCCTACTGAAGTGACGTGAGAC

MEKKKMVTQG NQEPTTTPDA MVQPFTTIPF PPPPQNGIPT EYGVPHTQDY AGQTSEHNLT
LYGSSQPHGE QSSNSPSNQN GSLTQTEGGA QTDGQQSQTQ SSENSESKST PKRLHVSNIP
FRFRDPDLRQ MFGQFGKILD VEIIFNERGS KGFGFVTFEN SADADRAREK LHGTVVEGRK
IEVNNATARV MTNKKMVTPY ANGWKLSPVV GAVYGPELYA ASSFQADVSL GNEAAVPLSG
RGGINTYIPL ISLPLVPGFP YPTAATTAAA FRGAHLRGRG RTVYGAVRAV PPTAIPAYPG
VVYQDGFYGA DLYGGYAAYR YAQPATATAA TAAAAAAAAY SDGYGRVYTA DPYHALAPAA
SYGVGAVASL YRGGYSRFAP Y

+32

ATGGAGAAAAAGAAAATGGTAACTCAGGGTAACCAGGAGCCAACAACAACTCCTGACGCAATGGTTCAG
CCTTTTACTACCATCCCATTCCCACCACCTCCACAGAACGGAATTCCCACAGAATATGGAGTGCCACAC
ACTCAGGACTATGCCGGCCAGACCAGTGAACATAACCTGACACTCTACGGGAGTTCACAGCCCCATGGA
GAACAGAGTAGCAATTCACCCAGCAACCAGAATGGATCTCTCACGCAGACAGAAGGTGGAGCACAGACA
GACGGACAACAGTCACAGACACAAAGTAGTGAAAATTCAGAGAGTAAATCCACGCCCAAGCGACTACAT
GTCTCTAATATTCCCTTCCGCTTCCGGGACCCTGACCTCCGGCAGATGTTTGGGCAGTTTGGCAAAATC
CTAGATGTGGAAATAATCTTTAATGAGCGCGGTTCCAAGGGATTCGGGTTCGTAACTTTCGAGAATAGT
GCTGATGCAGACAGGGCCAGGGAGAAATTGCACGGCACCGTGGTAGAGGGCCGTAAAATCGAGGTGAAT
AATGCTACCGCACGGGTCATGACCAACAAGAAGATGGTCACACCATATGCAAATGGCTGGAAGTTAAGC
CAGTAGTTGGAGCTGTGTACGGCCCTGAGTTATATGCAGCATCCAGCTTTCAAGCTGATGTGTCCCTAG
GCAATGAGGCGGCTGTGCCCTTGTCAGGAAGAGGGGGCATCAACACTTACATTCCTCTAATCAGTCTCC
CTTTAGTTCCTGGCTTCCCTTACCCAACTGCAGCCACCACAGCAGCCGCTTTCAGAGGAGCCCATCTGA
GGGGCAGAGGGCGGACAGTGTATGGTGCAGTGCGAGCGGTACCTCCAACAGCCATCCCCGCCTATCCAG
GTGTGGTTTACCAGGACGGATTTTACGGTGCTGACCTCTATATAGAATCTGCAAACTGCTTCAGATCAA
ACAGGGTGGATATGCAGCCTACAGATATGCACAGCCTGCTACTGCAACCGCAGCCACAGCTGCTGCAGC
CGCTGCAGCCGCTTACAGCGACGGTTACGGCAGGGTGTACACAGCTGACCCCTACCATGCCCTCGCCCC
TGCCGCCAGCTATGGAGTTGGCGCTGTGGCGAGTTTGTACCGAGGTGGCTACAGCCGATTTGCCCCCTA
CTGAAGTGACGTGA

MEKKKMVTQGNQEPTTTPDAMVQPFTTIPFPPPPQNGIPTEYGVPHTQDYAGQTSEHNLT
LYGSSQPHGEQSSNSPSNQNGSLTQTEGGAQTDGQQSQTQSSENSESKSTPKRLHVSNIP
FRFRDPDLRQMFGQFGKILDVEIIFNERGSKGFGFVTFENSADADRAREKLHGTVVEGRK
IEVNNATARVMTNKKMVTPYANGWKLSPVVGAVYGPELYAASSFQADVSLGNEAAVPLSG
RGGINTYIPLISLPLVPGFPYPTAATTAAAFRGAHLRGRGRTVYGAVRAVPPTAIPAYPG
VVYQDGFYGADLY IESANCFRSNRVDMQPTDMHSLLLQPQPQLLQPLQPLTATVTAGCTQ
LTPTMPSPLPPAMELALWRVCTEVATADLPPTEVT

-12+32

ATGGAGAAAAAGAAAATGGTAACTCAGGGTAACCAGGAGCCAACAACAACTCCTGACGCAATGGTTCAG
CCTTTTACTACCATCCCATTCCCACCACCTCCACAGAACGGAATTCCCACAGAATATGGAGTGCCACAC
ACTCAGGACTATGCCGGCCAGACCAGTGAACATAACCTGACACTCTACGGGAGTTCACAGCCCCATGGA
GAACAGAGTAGCAATTCACCCAGCAACCAGAATGGATCTCTCACGCAGACAGAAGGTGGAGCACAGACA
GACGGACAACAGTCACAGACACAAAGTAGTGAAAATTCAGAGAGTAAATCCACGCCCAAGCGACTACAT
GTCTCTAATATTCCCTTCCGCTTCCGGGACCCTGACCTCCGGCAGATGTTTGGGCAGTTTGGCAAAATC
CTAGATGTGGAAATAATCTTTAATGAGCGCGGTTCCAAGGGATTCGGGTTCGTAACTTTCGAGAATAGT
GCTGATGCAGACAGGGCCAGGGAGAAATTGCACGGCACCGTGGTAGAGGGCCGTAAAATCGAGGTGAAT
AATGCTACCGCACGGGTCATGACCAACAAGAAGATGGTCACACCATATGCAAATGGCTGGAAGTTAAGC
CCAGTAGTTGGAGCTGTGTACGGCCCTGAGTTATATGCAGCATCCAGCTTTCAAGCTGATGTGTCCCTA
GGCAATGAGGCGGCTGTGCCCTTGTCAGGAAGAGGGGGCATCAACACTTACATTCCTCTAATCATTCCT
GGCTTCCCTTACCCAACTGCAGCCACCACAGCAGCCGCTTTCAGAGGAGCCCATCTGAGGGGCAGAGGG
CGGACAGTGTATGGTGCAGTGCGAGCGGTACCTCCAACAGCCATCCCCGCCTATCCAGGTGTGGTTTAC
CAGGACAGATTTTACGGTGCTGACCTCTATATAGAATCTGCAAACTGCTTCAGATCAAACAGGGTGGAT
ATGCAGCCTACAGATATGCACAGCCTGCTACTGCAACCGCAGCCACAGCTGCTGCAGCCGCTGCAGCCG
CTTACAGCGACGGTTACGGCAGGGTGTACACAGCTGACCCCTACCATGCCCTCGCCCCTGCCGCCAGCT
ATGGAGTTGGCGCTGTGGCGAGTTTGTACCGAGGTGGCTACAGCCGATTTGCCCCCTACTGAAGTGACG
TGAGACAATCCCGCGGCCATGGCGGCCGGAGCA

MEKKKMVTQG NQEPTTTPDA MVQPFTTIPF PPPPQNGIPT EYGVPHTQDY AGQTSEHNLT
LYGSSQPHGE QSSNSPSNQN GSLTQTEGGA QTDGQQSQTQ SSENSESKST PKRLHVSNIP
FRFRDPDLRQ MFGQFGKILD VEIIFNERGS KGFGFVTFEN SADADRAREK LHGTVVEGRK
IEVNNATARV MTNKKMVTPY ANGWKLSPVV GAVYGPELYA ASSFQADVSL GNEAAVPLSG
RGGINTYIPL IIPGFPYPTA ATTAAAFRGA HLRGRGRTVY GAVRAVPPTA IPAYPGVVYQ
DRFYGADLYI ESANCFRSNR VDMQPTDMHS LLLQPQPQLL QPLQPLTATV TAGCTQLTPT
MPSPLPPAME LALWRVCTEV ATADLPPTEV T

-3a-12

ATGGAGAAAAAGAAAATGGTAACTCAGGGTAACCAGGAGCCAACAACAACTCCTGACGCAATGGTTCAG
CCTTTTACTACCATCCCATTCCCACCACCTCCACAGAACGGAATTCCCACAGAATATGGAGTGCCACAC
ACTCAGGCTATGCCGGCCAGACCAGTGAACATAACCTGACACTCTACGGGAGTTCACAGCCCCATGGAG
AACAGAGTAGCAATTCACCCAGCAACCAGAATGGATCTCTCACGACAGAAGGTGGAGCACAGACAGACG
ACAACAGTCACAGACACAAAGTAGTGAAAATTCAGAGAGTAAATCCACGCCCAAGCGACTACATGTCTC
TAATATTCCCTTCCGCTTCCGGGACCCTGACCTCCGGCAGATGTTTGGGCAGTTTGGCAAAATCCTAGA
TGTGGAAATAATCTTTAATGAGCGCGGTTCCAAGGGATTCGGGTTCGTAACTTTCGAGAATAGTGCTGA
TGCAGACAGGGCCAGGGAGAAATTGCACGGCACCGTGGTAGAGGGCCGTAAAATCGAGGTGAATAATGC
TACCGCACGGGTCATGACCAACAAGAAGATGGTCACACCATATGCAAATGGCTGGAAGTTAAGCCCAGT
AGTTGGAGCTGTGTACGGCCCTGAGTTATATGCAGCATCCAGCTTTCAAGCTGATGTGTCCCTAGGCAA
TGAGGCGGCTGTGCCCTTGTCAGGAAGAGGGGGCATCAACACTTACATTCCTCTAATCATTCCTGGCTT
CCCTTACCCAACTGCAGCCACCACAGCAGCCGCTTTCAAAGGAGCCCATCTGAGGGGCAGAGGGCGGAC
AGTGTATGGTGCAGTGCGAGCGGTACCTCCAACAGCCATCCCCGCCTATCCAGGTGTGGTTTACCAGGA
CGGATTTTACGGTGCTGACCTCTATGGTGGATATGCAGCCTACAGATATGCACAGCCTGCTACTGCAAC
CGCAGCCACAGCTGCTGCAGCCGCTGCAGCCGCTTACAGCGACGGTTACGGCAGGGTGTACACAGCTGA
CCCCTACCATGCCCTCGCCCCTGCCGCCAGCTATGGAGTTGGCGCTGTGGCGAGTTTGTACCGAGGTGG
CTACAGCCGATTTGCCCCCTACTGAAGTGACGTGA

MEKKKMVTQGNQEPTTTPDAMVQPFTTIPFPPPPQNGIPTEYGVPHTQDYAGQTSEHNLT
LYGSSQPHGEQSSNSPSNQNGSLTTEGGAQTDGQQSQTQSSENSESKSTPKRLHVSNIPF
RFRDPDLRQMFGQFGKILDVEIIFNERGSKGFGFVTFENSADADRAREKLHGTVVEGRKI
EVNNATARVMTNKKMVTPYANGWKLSPVVGAVYGPELYAASSFQADVSLGNEAAVPLSGR
GGINTYIPLIIPGFPYPTAATTAAAFRGAHLRGRGRTVYGAVRAVPPTAIPAYPGVVYQD
GFYGADLYGGYAAYRYAQPATATAATAAAAAAAAYSDGYGRVYTADPYHALAPAASYGVG
AVASLYRGGYSRFAPY

-12-40

ATGGAGAAAAAGAAAATGGTAACTCAGGGTAACCAGGAGCCAACAACAACTCCTGACGCAATGGTTCAG
CCTTTTACTACCATCCCATTCCCACCACCTCCACAGAACGGAATTCCCACAGAATATGGAGTGCCACAC
ACTCAGGACTATGCCGGCCAGACCAGTGAACATAACCTGACACTCTACGGGAGTTCACAGCCCCATGGA
GAACAGAGTAGCAATTCACCCAGCAACCAGAATGGATCTCTCACGCAGACAGAAGGTGGAGCACAGACA
GACGGACAACAGTCACAGACACAAAGTAGTGAAAATTCAGAGAGTAAATCCACGCCCAAGCGACTACAT

GTCTCTAATATTCCCTTCCGCTTCCGGGACCCTGACCTCCGGCAGATGTTTGGGCAGTTTGGCAAAATC
CTAGATGTGGAAATAATCTTTAATGAGCGCGGTTCCAAGGGATTCGGGTTCGTAACTTTCGAGAATAGT
GCTGATGCAGACAGGGCCAGGGAGAAATTGCACGGCACCGTGGTAGAGGGCCGTAAAATCGAGGTGAAT
AATGCTACCGCACGGGTCATGACCAACAAGAAGATGGTCACACCATATGCAAATGGCTGGAAGTTAAGC
CCAGTAGTTGGAGCTGTGTACGGCCCTGAGTTATATGCAGCATCCAGCTTTCAAGCTGATGTGTCCCTA
GGCAATGAGGCGGCTGTGCCCTTGTCAGGAAGAGGGGGCATCAACACTTACATTCCTCTAATCATTCCT
GGCTTCCCTTACCCAACTGCAGCCACCACAGCAGCCGCTTTCAGAGGAGCCCATCTGAGGGGCAGAGGG
CGGACAGTGTATGGTGCAGTGCGAGCGGTACCTCCAACAGCCATCCCCGCCTATCCAGGGGTGGATATG
CAGCCTACAGATATGCACAGCCTGCTACTGCAACCGCAGCCACAGCTGCTGCAGCCGCTGCAGCCGCTT
ACAGCGACGGTTACGGCAGGGTGTACACAGCTGACCCCTACCATGCCCTCGCCCCTGCCGCCAGCTATG
GAGTTGGCGCTGTGGCGAGTTTGTACCGAGGTGGCTACAGCCGATTTGCCCCCTACTGAAGTGACGTGA
GAC

MEKKKMVTQG NQEPTTTPDA MVQPFTTIPF PPPPQNGIPT EYGVPHTQDY AGQTSEHNLT
LYGSSQPHGE QSSNSPSNQN GSLTQTEGGA QTDGQQSQTQ SSENSESKST PKRLHVSNIP
FRFRDPDLRQ MFGQFGKILD VEIIFNERGS KGFGFVTFEN SADADRAREK LHGTVVEGRK
IEVNNATARV MTNKKMVTPY ANGWKLSPVV GAVYGPELYA ASSFQADVSL GNEAAVPLSG
RGGINTYIPL IIPGFPYPTA ATTAAAFRGA HLRGRGRTVY GAVRAVPPTA IPAYPGVDMQ
PTDMHSLLLQ PQPQLLQPLQ PLTATVTAGC TQLTPTMPSP LPPAMELALW RVCTEVATAD
LPPTEVT

-39

ATGGAGAAAAAGAAAATGGTAACTCAGGGTAACCAGGAGCCAACAACAACTCCTGACGCAATGGTTCAG
CCTTTTACTACCATCCCATTCCCACCACCTCCACAGAACGGAATTCCCACAGAATATGGAGTGCCACAC
ACTCAGGACTATGCCGGCCAGACCAGTGAACATAACCTGACACTCTACGGGAGTTCACAGCCCCATGGA
GAACAGAGTAGCAATTCACCCAGCAACCAGAATGGATCTCTCACGCAGACAGAAGGTGGAGCACAGACA
GACGGACAACAGTCACAGACACAAAGTAGTGAAAATTCAGAGAGTAAATCCACGCCCAAGCGACTACAT
GTCTCTAATATTCCCTTCCGCTTCCGGGACCCTGACCTCCGGCAGATGTTTGGGCAGTTTGGCAAAATC
CTAGATGTGGAAATAATCTTTAATGAGCGCGGTTCCAAGGGATTCGGGTTCGTAACTTTCGAGAATAGT
GCTGATGCAGACAGGGCCAGGGAGAAATTGCACGGCACCGTGGTAGAGGGCCGTAAAATCGAGGTGAAT
AATGCTACCGCACGGGTCATGACCAACAAGAAGATGGTCACACCATATGCAAATGGCTGGAAGTTAAGC
CCAGTAGTTGGAGCTGTGTACGGCCCTGAGTTATATGCAGCATCCAGCTTTCAAGCTGTTGTGTCCCTA
GGCAATGAGGCGGCTGTGCCCTTGTCAGGAAGAGGGGGCATCAACACTTACATTCCTCTAATCACCACC
ACAGCAGCCGCTTTCAGAGGAGCCCATCTGAGGGGCAGAGGGCGGACAGTGTATGGTGCAGTGCGAGCG
GTACCTCCAACAGCCATCCCCGCCTATCCAGGTGTGGTTTACCAGGACGGATTTTACGGTGCTGACCTC
TATGGTGGATATGCAGCCTACAGATATGCACAGCCTGCTACTGCAACCGCAGCCACAGCTGCTGCAGCC
GCTGCAGCCGCTTACAGCGACGGTTACGGCAGGGTGTACACAGCTGACCCCTACCATGCCCTCGCCCCT
GCCGCCAGCTATGGAGTTGGCGCTGTGGCGAGTTTGTACCGAGGTGGCTACAGCCGATTTGCCCCCTAC
TGAAGTGACGTGAGAC

MEKKKMVTQG NQEPTTTPDA MVQPFTTIPF PPPPQNGIPT EYGVPHTQDY AGQTSEHNLT
LYGSSQPHGE QSSNSPSNQN GSLTQTEGGA QTDGQQSQTQ SSENSESKST PKRLHVSNIP
FRFRDPDLRQ MFGQFGKILD VEIIFNERGS KGFGFVTFEN SADADRAREK LHGTVVEGRK
IEVNNATARV MTNKKMVTPY ANGWKLSPVV GAVYGPELYA ASSFQAVVSL GNEAAVPLSG
RGGINTYIPL ITTTAAAFRG AHLRGRGRTV YGAVRAVPPT AIPAYPGVVY QDGFYGADLY
GGYAAYRYAQ PATATAATAA AAAAAAYSDG YGRVYTADPY HALAPAASYG VGAVASLYRG
GYSRFAPY

-93

ATGGAGAAAAAGAAAATGGTAACTCAGGGTAACCAGGAGCCAACAACAACTCCTGACGCAATGGTTCAG
CCTTTTACTACCATCCCATTCCCACCACCTCCACAGAACGGAATTCCCACAGAATATGGAGTGCCACAC
ACTCAGGACTATGCCGGCCAGACCAGTGAACATAACCTGACACTCTACGGGAGTTCACAGCCCCATGGA
GAACAGAGTAGCAATTCACCCAGCAACCAGAATGGATCTCTCACGCAGACAGAAGGTGGAGCACAGACA
GACGGACAACAGTCACAGACACAAAGTAGTGAAAATTCAGAGAGTAAATCCACGCCCAAGCGACTACAT
GTCTCTAATATTCCCTTCCGCTTCCGGGACCCTGACCTCCGGCAGATGTTTGGGCAGTTTGGCAAAATC
CTAGATGTGGAAATAATCTTTAATGAGCGCGGTTCCAAGGTGAATAATGCTACCGCACGGGTCATGACC
AACAAGAAGATGGTCACACCATATGCAAATGGCTGGAAGTTAAGCCCAGTAGTTGGAGCTGTGTACGGC
CCTGAGTTATATGCAGCATCCAGCTTTCAAGCTGATGTGTCCCTAGGCAATGAGGCGGCTGTGCCCTTG
TCAGGAAGAGGGGGCATCAACACTTACATTCCTCTAATCAGTCTCCCTTTAGTTCCTGGCTTCCCTTAC
CCAACTGCAGCCACCACAGCAGCCGCTTTCAGAGGAGCCCATCTGAGGGGCAGAGGGCGGACAGTGTAT
GGTGCAGTGCGAGCGGTACCTCCAACAGCCATCCCCGCCTATCCAGGTGTGGTTTACCAGGACGGATTT
TACGGTGCTGACCTCTATGGTGGATATGCAGCCTACAGATATGCACAGCCTGCTACTGCAACCGCAGCC

ACAGCTGCTGCAGCCGCTGCAGCCGCTTACAGCGACGGTTACGGCAGGGTGTACACAGCTGACCCCTAC
CATGCCCTCGCCCCTGCCGCCAGCTATGGAGTTGGCGCTGTGGCGAGTTTGTACCGAGGTGGCTACA
GCCGATTTGCCCCCTACTGAAGTGACGTGAGAC

MEKKKMVTQGNQEPTTTPDAMVQPFTTIPFPPPPQNGIPTEYGVPHTQDYAGQTSEHNLT
LYGSSQPHGEQSSNSPSNQNGSLTTEGGAQTDGQQSQTQSSENSESKSTPKRLHVSNIPF
RFRDPDLRQMFGQFGKILDVEIIFNERGSKGFGFVTFENSADADRAREKLHGTVVEGRKI
EVNNATARVMTNKKMVTPYANGWKLSPVVGAVYGPELYAASSFQADVSLGNEAAVPLSGR
GGINTYIPLIIPGFPYPTAATTAAAFRGAHLRGRGRTVYGAVRAVPPTAIPAYPGVVYQD
GFYGADLYGGYAAYRYAQPATATAATAAAAAAAAYSDGYGRVYTADPYHALAPAASYGVG
AVASLYRGGYSRFAPY

-93+73
ATGGAGAAAAAGAAAATGGTAACTCAGGGTAACCAGGAGCCAACAACAACTCCTGACGCAATGGTTCAG
CCTTTTACTACCATCCCATTCCCACCACCTCCACAGAACGGAATTCCCACAGAATATGGAGTGCCACAC
ACTCAGGACTATGCCGGCCAGACCAGTGAACATAACCTGACACTCTACGGGAGTTCACAGCCCCATGGA
GAACAGAGTAGCAATTCACCCAGCAACCAGAATGGATCTCTCACGCAGACAGAAGGTGGAGCACAGACA
GACGGACAACAGTCACAGACACAAAGTAGTGAAAATTCAGAGAGTAAATCCACGCCCAAGCGACTACAT
GTCTCTAATATTCCCTTCCGCTTCCGGGACCCTGACCTCCGGCAGATGTTTGGGCAGTTTGGCAAAATC
CTAGATGTGGAAATAATCTTTAATGAGCGCGGTTCCAAGCATTTAGGGCCCTTCACTTGCCTCACTCTT
TCCATGGTAACTATACACAGTGCTGGCGATGGTGCCCGTTGGCGGTGAATAATGCTACCGCACGGGTCA
TGACCAACAAGAAGATGGTCACACCATATGCAAATGGCTGGAAGTTAAGCCCAGTAGTTGGAGCTGTGT
ACGGCCCTGAGTTATATGCAGCATCCAGCTTTCAAGCTGATGTGTCCCTAGGCAATGAGGCGGCTGTGC
CCTTGTCAGGAAGAGGGGGCATCAACACTTACATTCCTCTAATCAGTCTCCCTTTAGTTCCTGGCTTCC
CTTACCCAACTGCAGCCACCACAGCAGCCGCTTTCAGAGGAGCCCATCTGAGGGGCAGAGGGCGGACAG
TGTATGGTGCAGTGCGAGCGGTACCTCCAACAGCCATCCCCGCCTATCCAGGTGTGGTTTACCAGGACG
GATTTTACGGTGCTGACCTCTATGGTGGATATGCAGCCTACAGATATGCACAGCCTGCTACTGCAACCG
CAGCCACAGCTGCTGCAGCCGCTGCAGCCGCTTACAGCGACGGTTACGGCAGGGTGTACACAGCTGACC
CCTACCATGCCCTCGCCCCTGCCGCCAGCTATGGAGTTGGCGCTGTGGCGAGTTTGTACCGAGGTGGCT
ACAGCCGATTTGCCCCCTACTGAAGTGACGTGAGAC

MEKKKMVTQGNQEPTTTPDAMVQPFTTIPFPPPPQNGIPTEYGVPHTQDYAGQTSEHNLT
LYGSSQPHGEQSSNSPSNQNGSLTQTEGGAQTDGQQSQTQSSENSESKSTPKRLHVSNIP
FRFRDPDLRQMFGQFGKILDVEIIFNERGSKHLGPFTCLTLSMVTIHSAGDGARWR*IML
PHGS*PTRRWSHHMQMAGS*AQ*LELCTALSYMQHPAFKLMCP*AMRRLCPCQEEGASTL
TFL*SVSL*FLASLTQLQPPQQPLSEEPI*GAEGGQCMVQCERYLQQPSPPIQVWFTRTD
FTVLTSMVDMQPTDMHSLLLQPQPQLLQPLQPLTATVTAGCTQLTPTMPSPLPPAMELAL
WRVCTEVATADLPPTEVT

-93-12
ATGGAGAAAAAGAAAATGGTAACTCAGGGTAACCAGGAGCCAACAACAACTCCTGACGCAATGGTTCAG
CCTTTTACTACCATCCCATTCCCACCACCTCCACAGAACGGAATTCCCACAGAATATGGAGTGCCACAC
ACTCAGGACTATGCCGGCCAGACCAGTGAACATAACCTGACACTCTACGGGAGTTCACAGCCCCATGGA
GAACAGAGTAGCAATTCACCCAGCAACCAGAATGGATCTCTCACGCAGACAGAAGGTGGAGCACAGACA
GACGGACAACAGTCACAGACACAAAGTAGTGAAAATTCAGAGAGTAAATCCACGCCCAAGCGACTACAT
GTCTCTAATATTCCCTTCCGCTTCCGGGACCCTGACCTCCGGCAGATGTTTGGGCAGTTTGGCAAAATC
CTAGATGTGGAAATAATCTTTAATGAGCGCGGTTCCAAGGTGAATAATGCTACCGCACGGGTCATGACC
AACAAGAAGATGGTCACACCATATGCAAATGGCTGGAAGTTAAGCCCAGTAGTTGGAGCTGTGTACGGC
CCTGAGTTATATGCAGCATCCAGCTTTCAAGCTGATGTGTCCCTAGGCAATGAGGCGGCTGTGCCCTTG
TCAGGAAGAGGGGGCATCAACACTTACATTCCTCTAATCATTCCTGGCTTCCCTTACCCAACTGCAGCC
ACCACAGCAGCCGCTTTCAGAGGAGCCCATCTGAGGGGCAGAGGGCGGACAGTGTATGGTGCAGTGCGA
GCGGTACCTCCAACAGCCATCCCCGCCTATCCAGGTGTGGTTTACCAGGACGGATTTTACGGTGCTGAC
CTCTATGGTGGATATGCAGCCTACAGATATGCACAGCCTGCTACTGCAACCGCAGCCACAGCTGCTGCA
GCCGCTGCAGCCGCTTACAGCGACGGTTACGGCAGGGTGTACACAGCTGACCCCTACCATGCCCTCGCC
CCTGCCGCCAGCTATGGAGTTGGCGCTGTGGCGAGTTTGTACCGAGGTGGCTACAGCCGATTTGCCCCC
TACTGAAGTGACGTGAGAC

MEKKKMVTQGNQEPTTTPDAMVQPFTTIPFPPPPQNGIPTEYGVPHTQDYAGQTSEHNLT
LYGSSQPHGEQSSNSPSNQNGSLTQTEGGAQTDGQQSQTQSSENSESKSTPKRLHVSNIP
FRFRDPDLRQMFGQFGKILDVEIIFNERGSKVNNATARVMTNKKMVTPYANGWKLSPVVG
AVYGPELYAASSFQADVSLGNEAAVPLSGRGGINTYIPLIIPGFPYPTAATTAAAFRGAH

LRGRGRTVYGAVRAVPPTAIPAYPGVVYQDGFYGADLYGGYAAYRYAQPATATAATAAAA
AAAAYSDGYGRVYTADPYHALAPAASYGVGAVASLYRGGYSRFAPY

-93-12-40
ATGGAGAAAAAGAAAATGGTAACTCAGGGTAACCAGGAGCCAACAACAACTCCTGACGCAATGGTTCAG
CCTTTTACTACCATCCCATTCCCACCACCTCCACAGAACGGAATTCCCACAGAATATGGAGTGCCACAC
ACTCAGGACTATGCCGGCCAGACCAGTGAACATAACCTGACACTCTACGGGAGTTCACAGCCCCATGGA
GAACAGAGTAGCAATTCACCCAGCAACCAGAATGGATCTCTCACGCAGACAGAAGGTGGAGCACAGACA
GACGGACAACAGTCACAGACACAAAGTAGTGAAAATTCAGAGAGTAAATCCACGCCCAAGCGACTACAT
GTCTCTAATATTCCCTTCCGCTTCCGGGACCCTGACCTCCGGCAGATGTTTGGGCAGTTTGGCAAAATC
CTAGATGTGGAAATAATCTTTAATGAGCGCGGTTCCAAGGTGAATAATGCTACCGCACGGGTCATGACC
AACAAGAAGATGGTCACACCATATGCAAATGGCTGGAAGTTAAGCCCAGTAGTTGGAGCTGTGTACGGC
CCTGAGTTATATGCAGCATCCAGCTTTCAAGCTGATGTGTCCCTAGGCAATGAGGCGGCTGTGCCCTTG
TCAGGAAGAGGGGGCATCAACACTTACATTCCTCTAATCATTCCTGGCTTCCCTTACCCAACTGCAGCC
ACCACAGCAGCCGCTTTCAGAGGAGCCCATCTGAGGGGCAGAGGGCGGACAGTGTATGGTGCAGTGCGA
GCGGTACCTCCAACAGCCATCCCCGCCTATCCAGGGGTGGATATGCAGCCTACAGATATGCACAGCCTG
CTACTGCAACCGCAGCCACAGCTGCTGCAGCCGCTGCAGCCGCTTACAGCGACGGTTACGGCAGGGTGT
ACACAGCTGACCCCTACCATGCCCTCGCCCCTGCCGCCAGCTATGGAGTTGGCGCTGTGGCGAGTTTGT
ACCGAGGTGGCTACA GCCGATTTGCCCCCTACTGAAGTGACGTGAGAC

MEKKKMVTQGNQEPTTTPDAMVQPFTTIPFPPPPQNGIPTEYGVPHTQDYAGQTSEHNLT
LYGSSQPHGEQSSNSPSNQNGSLTQTEGGAQTDGQQSQTQSSENSESKSTPKRLHVSNIP
FRFRDPDLRQMFGQFGKILDVEIIFNERGSKVNNATARVMTNKKMVTPYANGWKLSPVVG
AVYGPELYAASSFQADVSLGNEAAVPLSGRGGINTYIPLIIPGFPYPTAATTAAAFRGAH
LRGRGRTVYGAVRAVPPTAIPAYPGVDMQPTDMHSLLLQPQPQLLQPLQPLTATVTAGCT
QLTPTMPSPLPPAMELALWRVCTEVATADLPPTEVT

-3b-12-40
ATGGAGAAAAAGAAAATGGTAACTCAGGGTAACCAGGAGCCAACAACAACTCCTGACGCAATGGTTCAG
CCTTTTACTACCATCCCATTCCCACCACCTCCACAGAACGGAATTCCCACAGAATATGGAGTGCCACAC
ACTCAGGACTATGCCGGCCAGACCAGTGAACATAACCTGACACTCTACGGGAGTTCACAGCCCCATGGA
GAACAGAGTAGCAATTCACCCAGCAACCAGAATGGATCTCTCACGCAGACAGAAGGTGGAGCACAGACA
GACGGACAACAGTCACAGACACAAAGTAGTGAAAATTCAGAGAGTAAATCCACGCCCAAGCGACTACAT
GTCTCTAATATTCCCTTCCGCTTCCGGGACCCTGACCTCCGGCAGATGTTTGGGTTTGGCAAAATCCTA
GATGTGGAAATAATCTTTAATGAGCGCGGTTCCAAGGGATTCGGGTTCGTAACTTTCGAGAATAGTGCT
GATGCAGACAGGGCCAGGGAGAAATTGCACGGCACCGTGGTAGAGGGCCGTAAAATCGAGGTGAATAAT
GCTACCGCACGGGTCATGACCAACAAGAAGATGGTCACACCATATGCAAATGGCTGGAAGTTAAGCCCA
GTAGTTGGAGCTGTGTACGGCCCTGAGTTATATGCAGCATCCAGCTTTCAAGCTGATGTGTCCCTAGGC
AATGAGGCGGCTGTGCCCTTGTCAGGAAGAGGGGGCATCAACACTTACATTCCTCTAATCATTCCTGGC
TTCCCTTACCCAACTGCAGCCACCACAGCAGCCGCTTTCAGAGGAGCCCATCTGAGGGGCAGAGGGCGG
ACAGTGTATGGTGCAGTGCGAGCGGTACCTCCAACAGCCATCCCCGCCTATCCAGGGGTGGATATGCAG
CCTACAGATATGCACAGCCTGCTACTGCAACCGCAGCCACAGCTGCTGCAGCCGCTGCAGCCGCTTACA
GCGACGGTTACGGCAGGGTGTACACAGCTGACCCCTACCATGCCCTCGCCCCTGCCGCCAGCTATGGAG
TTGGCGCTGTGGCGAGTTTGTACCGAGGTGGCTACAGCCGATTTGCCCCCTACTGAAGTGACGTGA

MEKKKMVTQGNQEPTTTPDAMVQPFTTIPFPPPPQNGIPTEYGVPHTQDYAGQTSEHNLT
LYGSSQPHGEQSSNSPSNQNGSLTQTEGGAQTDGQQSQTQSSENSESKSTPKRLHVSNIP
FRFRDPDLRQMFGFGKILDVEIIFNERGSKGFGFVTFENSADADRAREKLHGTVVEGRKI
EVNNATARVMTNKKMVTPYANGWKLSPVVGAVYGPELYAASSFQADVSLGNEAAVPLSGR
GGINTYIPLIIPGFPYPTAATTAAAFRGAHLRGRGRTVYGAVRAVPPTAIPAYPGVDMQP
TDMHSLLLQPQPQLLQPLQPLTATVTAGCTQLTPTMPSPLPPAMELALWRVCTEVATADL
PPTEVT

SI3. *De novo* sequenced nucleotide, and predicted amino acid sequences of PC12 cell Rbfox2 isoforms.

'Minor' sequence variations (vs. Genbank) are highlighted in purple. These may represent polymorphisms or technical variants (PCR/sequencing error).

-2-40+43

```
ATGGAGAAAAAGAAAATGGTAACTCAGGGTAACCAGGAGCCAACAACAACTCCTGACGCAATGGTTCAG
CCTTTTACTACCATCCCATTCCCACCACCTCCACAGAACGGAATTCCCACAGAATATGGAGTGCCACAC
ACTCAGGACTATGCCGGCCAGACCAGTGAACATAACCTGACACTCTACGGGAGTTCACAGCCCCATGGA
GAACAGAGTAGCAATTCACCCAGCAACCAGAATGGATCTCTCACGCAGACAGAAGGTGGAGCACAGACA
GACGGACAACAGTCACAGACACAAAGTAGTGAAAATTCAGAGAGTAAATCCACGCCCAAGCGACTACAT
GTCTCTAATATTCCCTTCCGCTTCCGGGACCCTGACCTCCGGCAGATGTTTGGGCAGTTTGGCGAAATC
CTAGATGTGAAAATAATCTTTAATGAGCGCGGTTCCAAGGGATTCGGGTTCGTAACTTTCGAGAATAGT
GCTGATGCAGACAGGGCCAGGGAGAAATTGCACGGCACCGTGGTAGAGGGCCGTAAAATCGAGGTGAAT
AATGCTACCGCACGGGTCATGACCAACAAGAAGATGGTCACACCATATGCAAATGGCTGGAAGTTAAGC
CCAGTAGTTGGAGCTGTGTACGGCCCTGAGTTATATGCAGCATCCAGCTTTCAAGCTGATGTGTCCCCTA
GGCAATGAGGCGGCTGTGCCCTTGTCAGGAAGAGGGGGCATCAACACTTACATTCCTCTAATCAGTCTC
CCTTTAGTTCCTGGCTTCCCTTACCCAACTGCAGCCACCACAGCAGCCGCTTTCAGAGGAGCCCATCTG
AGGGGCAGAGGGCGGACAGTGTATGGTAGTGCGAGCGGTACCTCCAACAGCCATCCCCGCCTATCCAGG
AATAGTGTTACAGGAACCAATCATTAGCGCTAAAATACCTCAGGGTGGATATGCAGCCTACAGATATGC
ACAGCCTGCTACTGCAACCGCAGCCACAGCTGCTGCAGCCGCTGCAGCCGCTTACAGCGACGGTTACGG
CAGGGTGTACACAGCTGACCCCTACCATGCCCTCGCCCCTGCCGCCAGCTATGGAGTTGGCGCTGTGGC
GAGTTTGTACCGAGGTGGCTACAGCCGATTTGCCCCCTACTGAAGTGACGTGAGAC
```

GTAG -2bp microexon (GC)

```
MEKKKMVTQG NQEPTTTPDA MVQPFTTIPF PPPPQNGIPT EYGVPHTQDY AGQTSEHNLT
LYGSSQPHGE QSSNSPSNQN GSLTQTEGGA QTDGQQSQTQ SSENSESKST PKRLHVSNIP
FRFRDPDLRQ MFGQFGEILD VKIIFNERGS KGFGFVTFEN SADADRAREK LHGTVVEGRK
IEVNNATARV MTNKKMVTPY ANGWKLSPVV GAVYGPELYA ASSFQADVSL GNEAAVPLSG
RGGINTYIPL ISLPLVPGFP YPTAATTAAA FRGAHLRGRG RTVYGSASGT SNSHPRLSRN
SVTGTNH
```

Non-variant

```
ATGGAGAAAAAGAAAATGGTAACTCAGGGTAACCAGGAGCCAACAACAACTCCTGACGCAATGGTTCAG
CCTTTTACTACCATCCCATTCCCACCACCTCCACAGAACGGAATTCCCACAGAATATGGAGTGCCACAC
ACTCAGGACTATGCCGGCCAGACCAGTGAACATAACCTGACACTCTACGGGAGTTCACAGCCCCATGGA
GAACAGAGTAGCAATTCACCCAGCAACCAGAATGGATCTCTCACGCAGACAGAAGGTGGAGCACAGACA
GACGGACAACAGTCACAGACACAAAGTAGTGAAAATTCAGAGAGTAAATCCACGCCCAAGCGACTACAT
GTCTCTAATATTCCCTTCCGTTTCCGGGACCCTGACTTCCGGCAGATGTTTGGGCAGTTTGGCAAAATC
CTAGATGTGGAAATAATCTTTAATGAGCGCGGTCCCAAGGGATTCGGGTTCGTAACTTTCGAGAATAGT
GCTGATGCAGACAGGGCCAGGGAGAAATTGCACGGCACCGTGGTAGAGGGCCGTAAAATCGAGGTGAAT
AATGCTACCGCACGGGTCATGACCAACAAGAAGATGGTCACACCATATGCAAATGGCTGGAAGTTAAGC
CCAGTAGTTGGAGCTGTGTACGGCCCTGAGTTATATGCAGCATCCAGCTTTCAAGCTGATGTGTCCCCTA
GGCAATGAGGCGGCTGTGCCCTTGTCAGGAAGAGGGGGCATCAACACTTACATTCCTCTAATCAGTCTC
CCTTTAGTTCCTGGCTTCCCTTACCCAACTGCAGCCACCACAGCAGCCGCTTTCAGAGGAGCCCATCTG
AGGGGCAGAGGGCGGACAGTGTATGGTGCAGTGCGAGCGGTACCTCCAACAGCCATCCCCGCCTATCCA
GGTGTGGTTTACCAGGACGGATTTTACGGTGCTGACCTCTATGGTGGATATGCAGCCTACAGATATGCA
CAGCCTGCTACTGCAACCGCAGCCACAGCTGCTGCAGCCGCTGCAGCCGCTTACAGCGACGGTTACGGC
AGGGTGTACACAGCTGACCCCTACCATGCCCTCGCCCCTGCCGCCAGCTATGGAGTTGGCGCTGTGGCG
AGTTTGTACCGAGGTGGCTACAGCCGATTTGCCCCCTACTGAAGTGACGTGAGAC
```

```
MEKKKMVTQG NQEPTTTPDA MVQPFTTIPF PPPPQNGIPT EYGVPHTQDY AGQTSEHNLT
LYGSSQPHGE QSSNSPSNQN GSLTQTEGGA QTDGQQSQTQ SSENSESKST PKRLHVSNIP
FRFRDPDFRQ MFGQFGKILD VEIIFNERGP KGFGFVTFEN SADADRAREK LHGTVVEGRK
IEVNNATARV MTNKKMVTPY ANGWKLSPVV GAVYGPELYA ASSFQADVSL GNEAAVPLSG
RGGINTYIPL ISLPLVPGFP YPTAATTAAA FRGAHLRGRG RTVYGAVRAV PPTAIPAYPG
VVYQDGFYGA DLYGGYAAYR YAQPATATAA TAAAAAAAY SDGYGRVYTA DPYHALAPAA
SYGVGAVASL YRGGYSRFAP Y
```

-3b-12

ATGGAGAAAAAGAAAATGGTAACTCAGGGTAACCAGGAGCCAACAACAACTCCTGACGCAATGGTTCAG
CCTTTTACTACCATCCCATTCCCACCACCTCCACAGAACGGAATTCCCACAGAATATGGAGTGCCACAC
ACTCAGGACTATGCCGGCCAGACCAGTGAACATAACCTGACACTCTACGGGAGTTCACAGCCCCATGGA
GAACAGAGTAGCAATTCACCCAGCAACCAGAATGGATCTCTCACGCAGACAGAAGGTGGAGCACAGACA
GACGGACAACAGTCACAGACACAAAGTAGTGAAAATTCAGAGAGTAAATCCACGCCCAAGCGACTACAT
GTCTCTAATATTCCCTTCCGCTTCCGGGACCCTGACCTCCGGCAGATGTTTGGGTTTGGCAAAATCCTA
GATGTGGAAATAATCTTTAATGAGCGCGGTTCCAAGGGATTCGGGTTCGTAACTTTCGAGAATAGTGCT
GATGCAGACAGGGCCAGGGAGAAATTGCACGGCACCGTGGTAGAGGGCCGTAAAATCGAGGTGAATAAT
GCTACCGCACGGGTCATGACCAACAAGAAGATGGTCACACCATATGCAAATGGCTGGAAGTTAAGCCCA
GTAGTTGGAGCTGTGTACGGCCCTGAGTTATATGCAGCATCCAGCTTTCAAGCTGATGTGTCCCTAGGC
AATGAGGCGGCTGTGCCCTTGTCAGGAAGAGGGGGCATCAACACTTACATTCCTCTAATCATTCCTGGC
TTCCCTTACCCAACTGCAGCCACCACAGCAGCCGCTTTCAGAGGAGCCCATCTGAGGGGCAGAGGGCGG
ACAGTGTATGGTGCAGTGCGAGCGGTACCTCCAACAGCCATCCCCGCCTATCCAGGTGTGGTTTACCAG
GACGGATTTTACGGTGCTGACCTCTATGGTGGATATGCAGCCTACAGATATGCACAGCCTGCTACTGCA
ACCGCAGCCACAGCTGCTGCAGCCGCTGCAGCCGCTTACAGCGACGGTTACGGCAGGGTGTACACAGCT
GACCCCTACCATGCCCTCGCCCCTGCCGCCAGCTATGGAGTTGGCGCTGTGGCGAGTTTGTACCGAGGT
GGCTACAGCCGATTTGCCCCCTACTGAAGTGACGTGAGAC

MEKKKMVTQG NQEPTTTPDA MVQPFTTIPF PPPPQNGIPT EYGVPHTQDY AGQTSEHNLT
LYGSSQPHGE QSSNSPSNQN GSLTQTEGGA QTDGQQSQTQ SSENSESKST PKRLHVSNIP
FRFRDPDLRQ MFGFGKILDV EIIFNERGSK GFGFVTFENS ADADRAREKL HGTVVEGRKI
EVNNATARVM TNKKMVTPYA NGWKLSPVVG AVYGPELYAA SSFQADVSLG NEAAVPLSGR
GGINTYIPLI IPGFPYPTAA TTAAAFRGAH LRGRGRTVYG AVRAVPPTAI PAYPGVVYQD
GFYGADLYGG YAAYRYAQPA TATAATAAAA AAAAYSDGYG RVYTADPYHA LAPAASYGVG
AVASLYRGGY SRFAPY

-12

ATGGAGAAAAAGAAAATGGTAACTCAGGGTAACCAGGAGCCAACAACAACTCCTGACGCAATGGTTCAG
CCTTTTACTACCATCCCATTCCCACCACCTCCACAGAACGGAATTCCCACAGAATATGGAGTGCCACAC
ACTCAGGACTATGCCGGCCAGACCAGTGAACATAACCTGACACTCTACGGGAGTTCACAGCCCCATGGA
GAACAGAGTAGCAATTCACCCAGCAACCAGAATGGATCTCTCACGCAGACAGAAGGTGGAGCACAGACA
GACGGACAACAGTCACAGACACAAAGTAGTGAAAATTCAGAGAGTAAATCCACGCCCAAGCGACTACAT
GTCTCTAATATTCCCTTCCGCTTCCGGGACCCTGACCTCCGGCAGATGTTTGGGCAGTTTGGCAAAATC
CTAGATGTGGAAATAATCTTTAATGAGCGCGGTTCCAAGGGATTCGGGTTCGTAACTTTCGAGAATAGT
GCTGATGCAGACAGGGCCAGGGAGAAATTGCACGGCACCGTGGTAGAGGGCCGTAAAATCGAGGTGAAT
AATGCTACCGCACGGGTCATGACCAACAAGAAGATGGTCACACCATATGCAAATGGCTGGAAGTTAAGC
CCAGTAGTTGGAGCTGTGTACGGCCCTGAGTTATATGCAGCATCCAGCTTTCAAGCTGATGTGTCCCTA
GGCAATGAGGCGGCTGTGCCCTTGTCAGGAAGAGGGGGCATCAACACTTACATTCCTCTAATCATTCCT
GGCTTCCCTTACCCAACTGCAGCCACCACAGCAGCCGCTTTCAGAGGAGCCCATCTGAGGGGCAGAGGG
CGGACAGTGTATGGTGCAGTGCGAGCGGTACCTCCAACAGCCATCCCCGCCTATCCAGGTGTGGTTTAC
CAGGACGGATTTTACGGTGCTGACCTCTATGGTGGATATGCAGCCTACAGATATGCACAGCCTGCTACT
GCAACCGCAGCCACAGCTGCTGCAGCCGCTGCAGCCGCTTACAGCGACGGTTACGGCAGGGTGTACACA
GCTGACCCCTACCATGCCCTCGCCCCTGCCGCCAGCTATGGAGTTGGCGCTGTGGCGAGTTTGTACCGA
GGTGGCTACAGCCGATTTGCCCCCTACTGAAGTGACGTGAGAC

MEKKKMVTQG NQEPTTTPDA MVQPFTTIPF PPPPQNGIPT EYGVPHTQDY AGQTSEHNLT
LYGSSQPHGE QSSNSPSNQN GSLTQTEGGA QTDGQQSQTQ SSENSESKST PKRLHVSNIP
FRFRDPDLRQ MFGQFGKILD VEIIFNERGS KGFGFVTFEN SADADRAREK LHGTVVEGRK
IEVNNATARV MTNKKMVTPY ANGWKLSPVV GAVYGPELYA ASSFQADVSL GNEAAVPLSG
RGGINTYIPL IIPGFPYPTA ATTAAAFRGA HLRGRGRTVY GAVRAVPPTA IPAYPGVVYQ
DGFYGADLYG GYAAYRYAQP ATATAATAAA AAAAAYSDGY GRVYTADPYH ALAPAASYGV
GAVASLYRGG YSRFAPY

SI4. Alignment of predicted protein sequence of rat brain Rbfox2 isoforms.

```
CLUSTAL O(1.2.4) multiple sequence alignment

NONV          MEKKKMVTQGNQEPTTTPDAMVQPFTTIPFPPPPQNGIPTEYGVPHTQDYAGQTSEHNLT
1240          MEKKKMVTQGNQEPTTTPDAMVQPFTTIPFPPPPQNGIPTEYGVPHTQDYAGQTSEHNLT
              ************************************************************

NONV          LYGSSQPHGEQSSNSPSNQNGSLTQTEGGAQTDGQQSQTQSSENSESKSTPKRLHVSNIP
1240          LYGSSQPHGEQSSNSPSNQNGSLTQTEGGAQTDGQQSQTQSSENSESKSTPKRLHVSNIP
              ************************************************************

NONV          FRFRDPDLRQMFGQFGKILDVEIIFNERGSKGFGFVTFENSADADRAREKLHGTVVEGRK
1240          FRFRDPDLRQMFGQFGKILDVEIIFNERGSKGFGFVTFENSADADRAREKLHGTVVEGRK
              ************************************************************

NONV          IEVNNATARVMTNKKMVTPYANGWKLSPVVGAVYGPELYAASSFQADVSLGNEAAVPLSG
1240          IEVNNATARVMTNKKMVTPYANGWKLSPVVGAVYGPELYAASSFQADVSLGNEAAVPLSG
              ************************************************************

NONV          RGGINTYIPLISLPLVPGFPYPTAATTAAAFRGAHLRGRGRTVYGAVRAVPPTAIPAYPG
1240          RGGINTYIPLI----IPGFPYPTAATTAAAFRGAHLRGRGRTVYGAVRAVPPTAIPAYPG
              ***********    :********************************************

NONV          VVYQDGFYGADLYGGYAA-YRYAQPATATAATAAAAAAA--------AYSDGYGRVYTAD
1240          VDMQPTDMHSLLLQPQPQLLQPLQPLTATVTAGCTQLTPTMPSPLPPAMELALWRVCTEV
              *  *     : *        :  ** ***.::..:  :         * . .  ** *

NONV          PYHALAPAASYGVGAVASLYRGGYSRFAPY
1240          ATADLPPTEVT-------------------
                  * *:
```

NONV = non-variant isoform
1240 = -12-40 isoform

SI5. Comparison of protein domains between 'non-variant' and -12-40 isoforms.

Non-variant

```
MEKKKMVTQG NQEPTTTPDA MVQPFTTIPF PPPPQNGIPT EYGVPHTQDY AGQTSEHNLT
LYGSSQPHGE QSSNSPSNQN GSLTQTEGGA QTDGQQSQTQ SSENSESKST PKRLHVSNIP
FRFRDPDLRQ MFGQFGKILD VEIIFNERGS KGFGFVTFEN SADADRAREK LHGTVVEGRK
IEVNNATARV MTNKKMVTPY ANGWKLSPVV GAVYGPELYA ASSFQADVSL GNEAAVPLSG
RGGINTYIPL ISLPLVPGFP YPTAATTAAA FRGAHLRGRG RTVYGAVRAV PPTAIPAYPG
VVYQDGFYGA DLYGGYAAYR YAQPATATAA TAAAAAAAAY SDGYGRVYTA DPYHALAPAA
SYGVGAVASL YRGGYSRFAP Y
```

-12-40

```
MEKKKMVTQG NQEPTTTPDA MVQPFTTIPF PPPPQNGIPT EYGVPHTQDY AGQTSEHNLT
LYGSSQPHGE QSSNSPSNQN GSLTQTEGGA QTDGQQSQTQ SSENSESKST PKRLHVSNIP
FRFRDPDLRQ MFGQFGKILD VEIIFNERGS KGFGFVTFEN SADADRAREK LHGTVVEGRK
IEVNNATARV MTNKKMVTPY ANGWKLSPVV GAVYGPELYA ASSFQADVSL GNEAAVPLSG
RGGINTYIPL IIPGFPYPTA ATTAAAFRGA HLRGRGRTVY GAVRAVPPTA IPAYPGVDMQ
PTDMHSLLLQ PQPQLLQPLQ PLTATVTAGC TQLTPTMPSP LPPAMELALW RVCTEVATAD
LPPTEVT
```

RLH RRM domain
GRG CTD domain
I DMQ variant CTD domain

Table S1. Oligonucleotide sequences and use

Name	Method	Sequence (5'->3')
ACTBF	PCR	TCATGCCATCCTGCGTCTGGACCT
ACTBR	PCR	CCGGACTCATCGTACTCCTGCTTG
RBFF1	PCR	ATGGCGGAAGGCGGCCAGGC
RBFR3	PCR	GTCTCACGTCACTTCAGTAGG
RBFF6	PCR	CTGCTTCTTCTGGTTTATGGAG
KozRBFF6H	Expression construct PCR	GCATAAGCTTCTGCTTCTTCTGGATTATGGAG
RBFR3B	Expression construct PCR	GTATGGATCCGTCTCACGTCACTTCAGTAGG
EGR1F2	QPCR	GAACAACCCTACGAGCACCT
EGR1R1	QPCR	TTTGGCTGGGATAACTTGTC
18SF1	QPCR	GGGAGGTAGTGACGAAAAATAACAAT
18SR1	QPCR	TTGCCCTCCAATGGATCCT
18SRT	18S RT-primer	GAGCTGGAATTACCGCGGCT
CAVF1	PCR	GCTACCTGGACTGGATCACC
CAVR1	PCR	TCACCCTCGATGTCACCTCC
CAVF2	PCR	GATGATCCTGAAGCTCATTGC
CAVR2	PCR	TTGATGAAGGTCCACAGCAGG
ENAHF1	PCR	AACCAACCAGAAAACCTTGG
ENAHR1	PCR	TGCTTCAGCCTGTCATAGTC
GRINF1	PCR	TCCACCTGAGTTTCCTTCGC
GRINR1	PCR	GAACCACATCATCCTGCTGG

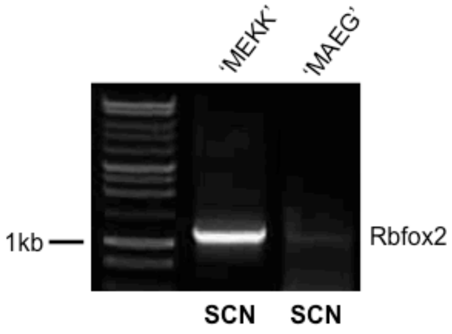

Fig.S1. Differential expression of MEKK and MAEG Rbfox2 isoforms in rat suprachiasmatic nucleus (SCN). Representative agarose gel electrophoresis image of RT-PCR analysis using forward primers directed against two different Rbfox2 start sites. PCR product size is estimated against the Promega 1kb ladder (left lane).

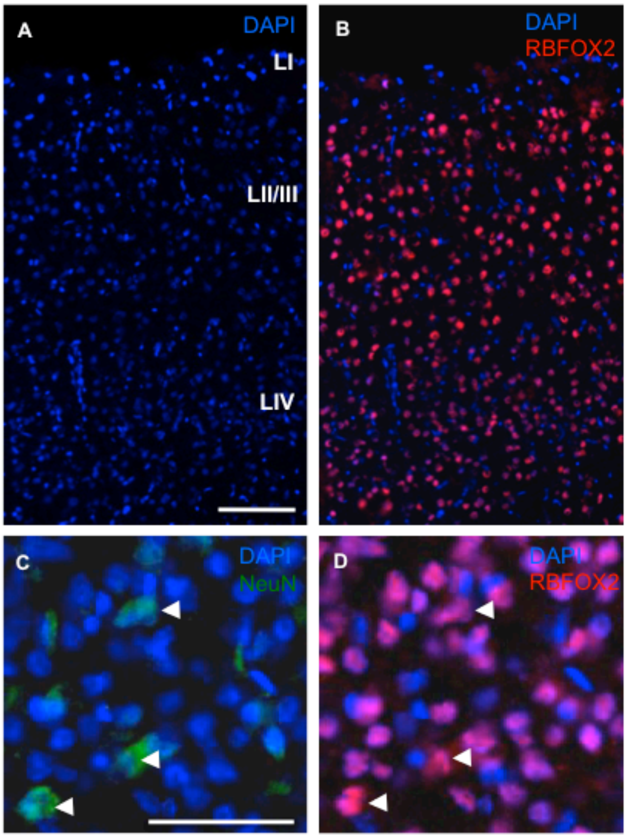

Fig.S2. Abundant RBFOX2 immunoreactivity in adult rat brain. Representative fluorescence microscopic images of male PN50 brain illustrating two aspects of RBFOX2 protein distribution. **A&B**. Abundant expression of RBFOX2 in neurons of the parietal cortex. **C &D**. Levels of RBFOX2 are similar in NeuN+ve and NeuN-ve neurons within the suprachiasmatic nucleus (SCN). Arrowheads indicate the position of three NeuN+ve neurons in a central region of the SCN in which levels of RBFOX2 are similar to adjacent NeuN-ve neurons. Abbreviations: DAPI, 4',6-diamidino-2-phenylindole; LI,LII,LIII,LIV, cortical layers. Scale bars: A&B, 100μm; C&D, 50μm.

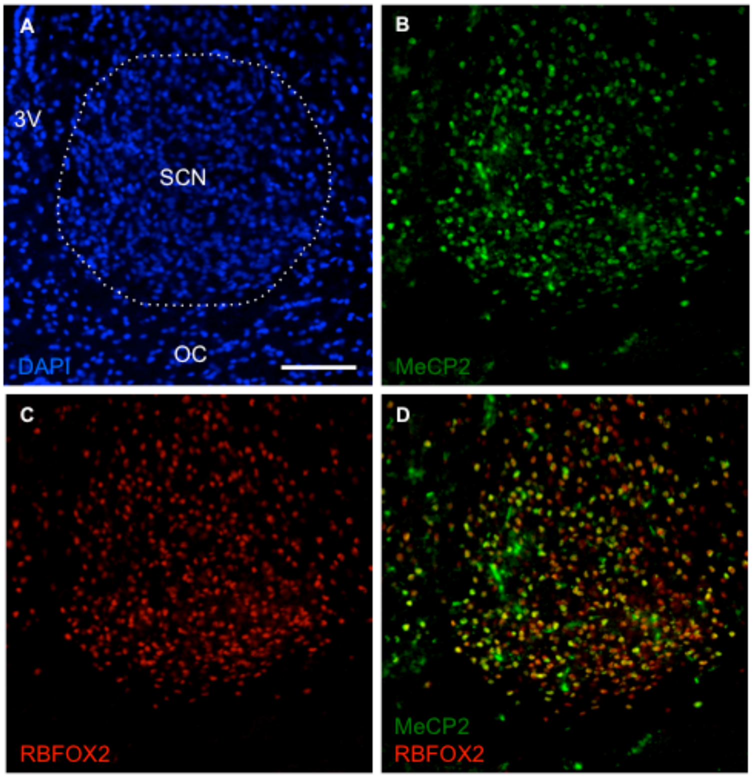

Fig.S3. RBFOX2 is co-localized with MeCP2 in rat suprachiasmatic nucleus (SCN) neurons. Representative fluorescence microscopic images of male PN50 brain showing the distribution of RBFOX2 immunoreactivity in neurons with respect to MeCP2. Note that the levels of RBFOX2 and MeCP2 immunoreactivity vary between individual neurons but these two proteins are extensively co-localized. Abbreviations: 3V, third ventricle; OC, optic chiasm; DAPI, 4',6-diamidino-2-phenylindole; MeCP2, methyl CpG-binding protein 2. Scale bar: A-C = 50µm.

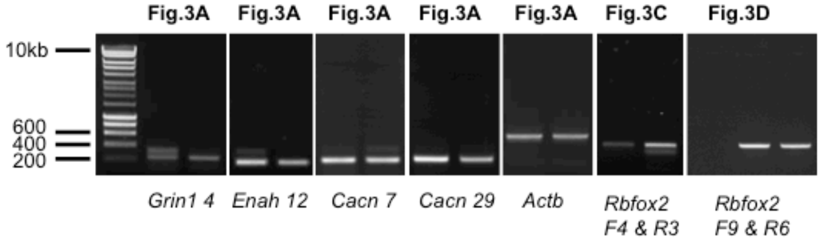

Fig.S4. Differential expression of RBFOX-target exons in rat brain cortex and suprachiasmatic nucleus. Representative agarose gel electrophoresis images of RT-PCR analysis. Images are similar to Figures 3A, 3C and 3D but show full-length gels (200bp to 10kb) for each of the PCR products analysed in Fig.3. Numbers after the gene symbols (eg.4, F4) refer to exon numbers, and primers, respectively. *Cacn* = *Cacna1c*. Note that due to slightly different conditions in each electrophoresis run, the product positions vary slightly between gels.
